# OsWRKY53‐OsGT1 Module Regulates Rice Tiller Development and Is Involved in Fine‐Tuning Strigolactone Signaling

**DOI:** 10.1111/pbi.70578

**Published:** 2026-02-05

**Authors:** Jiaqi Tang, Guilong Zhao, Jiangli Yang, Zhuo Chen, Zhipeng Hong, Xin Jin, Ziming Qiu, Zhenyu Wang, Xiufeng Li, Jijun Yan, Changhua Liu, Weiqiang Li, Jinfang Chu, Yuanhu Xuan, Xiaojie Tian, Qingyun Bu

**Affiliations:** ^1^ State Key Laboratory of Black Soils Conservation and Utilization, Northeast Institute of Geography and Agroecology Chinese Academy of Sciences Harbin China; ^2^ College of Advanced Agriculture and Ecological Environment Heilongjiang University Harbin China; ^3^ College of Horticulture Fujian Agriculture and Forestry University Fuzhou China; ^4^ University of Chinese Academy of Sciences Beijing China; ^5^ State Key Laboratory of Seed Innovation, National Center for Plant Gene Research (Beijing) Institute of Genetics and Developmental Biology, Chinese Academy of Sciences Beijing China; ^6^ State Key Laboratory of Elemento‐Organic Chemistry and Department of Plant Protection, National Pesticide Engineering Research Center, College of Chemistry Nankai University Tianjin China

**Keywords:** D53, OsTB1, OsWRKY53, rice, strigolactone, tiller

## Abstract

Plant architecture, including plant height, tiller number, and leaf angle, is a critical determinant of rice yield. However, few genes have been identified that simultaneously regulate these traits and hold breeding value. We have previously shown that OsWRKY53 regulates the plant height and leaf angle via BR signalling. Here, we establish OsWRKY53 as a novel negative regulator of tillering in rice. The *oswrky53* mutant exhibits a semi‐dwarf stature coupled with increased tiller number, representing a promising agronomic combination. Genetic and molecular analyses reveal that OsWRKY53 acts as a direct transcriptional activator of *OsTB1*, thereby suppressing tiller formation. In addition, we found OsWRKY53 physically interacts with OsGT1, and their cooperative action synergistically enhances *OsTB1* expression and suppresses tiller number. Intriguingly, the *oswrky53* mutant exhibits reduced sensitivity to Strigolactone (SL) and increased SL contents. We further demonstrate that SL promotes degradation of OsWRKY53, and D53 interacts with and stabilises the OsWRKY53. Simultaneously, OsWRKY53 negatively regulates SL biosynthesis, enabling OsWRKY53 to function as a fine‐tuning regulator in the SL signalling pathway. Furthermore, OsGT1 exhibits subspecies‐specific regulation, with *indica* accessions carrying the *OsGT1* ^*581T*^ allele showing significantly enhanced tillering capacity compared to *japonica* varieties. These findings collectively reveal the mechanism by which OsWRKY53 regulates the formation of tillers in rice, providing new genetic targets for semi‐dwarf and high‐tillering rice breeding.

## 1 Introduction

Rice ( *Oryza sativa* ) is a vital global food crop, essential for agricultural productivity and food security. Plant architecture, especially tiller number, plant height, and leaf angle significantly influences grain yield in rice. Since the 1960s, the widespread cultivation of dwarf rice and wheat varieties, known as the Green Revolution, has substantially boosted global food production. In rice, the short stature of Green Revolution Varieties results from a mutation in the *sd1* gene, which encodes GA20ox‐2 oxidase involved in the biosynthesis of gibberellin (GA) (Sasaki et al. 2002). Additionally, an allelic variant of the strigolactone (SL) biosynthesis gene *HTD1*/*DWARF17* (*HTD1*/*D17*) contributes to increased tiller number and has been co‐selected with *sd1* in *indica* rice since the Green Revolution. *HTD1* played a crucial role in optimising plant architecture and is regarded as a “Green Revolution companion gene” (Wang et al. 2020). These genetic advances have significantly enhanced yield and optimised agronomic traits in modern rice.

Plant hormones, including GA, brassinosteroid (BR), and SL, are known to simultaneously regulate both plant height and tillering (Liu et al. 2018; Zhang et al. 2025). However, none of their signalling components can replicate the beneficial effects of the “Green Revolution gene” *sd1*. A key limitation is that genetic perturbations in these hormone pathways often disrupt other essential yield‐related traits, rendering them unsuitable for practical breeding. For example, GA signalling mutants such as *gid1* and *slr1‐d* exhibit dwarfism and increased tillering but also impaired fertility or even complete sterility (Liao et al. 2019; Sakata et al. 2014; Ueguchi‐Tanaka et al. 2005). Similarly, BR‐related mutants like *d61‐2* (defective in *OsBRI1*) and *OsBZR1‐RNAi* show dwarf phenotypes along with reduced tillering (Fang et al. 2020). In contrast, mutants defective in SL biosynthesis or signalling, such as the rice dwarf mutants (*d3*, *d14*, *d10*, *d17*, *d27*), consistently exhibit excessive tillering phenotypes (Wang and Li 2006; Al‐Babili and Bouwmeester 2015). These pleiotropic effects are often detrimental to rice yield. Therefore, identifying key regulatory genes and elucidating their mechanisms in controlling plant architecture will provide essential genetic resources and mechanistic insights to advance rice breeding programs.

SLs, a class of carotenoid‐derived plant hormones, inhibit tiller bud outgrowth across diverse plant species. In rice, SL perception is mediated by the receptor DWARF14 (D14), an α/β hydrolase. Upon activation, D14 interacts with the F‐box protein DWARF3 (D3) to form the D14‐SCF^D3^ complex (Shabek et al. 2018; Yao et al. 2016). This complex facilitates the ubiquitination and degradation of key targets, including the transcriptional repressor DWARF53 (D53), ultimately leading to tiller suppression (Jiang et al. 2013; Zhou et al. 2013; Shabek et al. 2018). D53 directly interacts with OsIPA1 to inhibit its transcriptional activity. Furthermore, OsIPA1 can directly bind to the *D53* promoter and upregulate its expression, establishing a negative feedback loop that fine‐tunes SL signalling (Jiao et al. 2010; Miura et al. 2010; Wang et al. 2017; Song et al. 2017; Zhang et al. 2017).

The *OsTB1*/*OsFC1* gene encodes a TCP transcription factor that acts as a central regulator of tiller formation, functioning both within the SL pathway and serving as a hub that integrates multiple signalling pathways (Takeda et al. 2003; Aguilar‐Martı'nez et al. 2007; Miura et al. 2010; Martın‐Trillo et al. 2011; Gonzalez‐Grandio et al. 2013; Braun et al. 2012). OsIPA1 regulates *OsTB1* expression to suppress rice tillering (Lu et al. 2013). OsMADS57 interacts with OsTB1, which alleviates the inhibition of OsMADS57 on *D14*, thereby repressing rice tillering (Guo et al. 2013). Moreover, SL and BR pathways antagonistically regulate tillering by modulating the stability of the D53‐OsBZR1 complex, which in turn regulates the expression of *OsTB1* (Fang et al. 2020). Beyond these pathways, OsGT1/OsHOX12 mediates crosstalk between SL and abscisic acid (ABA) in tillering control (Gonzalez‐Grandio et al. 2017; Liu et al. 2020). OsTB1 directly binds to the *OsGT1* promoter to enhance ABA biosynthesis, further suppressing tiller number (Kumar et al. 2021). Although OsTB1 plays a pivotal role in tiller formation, its involvement in additional regulatory pathways remains to be explored.

OsWRKY53 has been implicated in diverse physiological processes, including the regulation of plant architecture, grain size, and multiple stress responses (Hu et al. 2016; Tian et al. 2017; Yuan et al. 2020; Xie et al. 2021; Gao et al. 2021; Tian et al. 2021; Tang et al. 2022; Yu et al. 2023; Yang et al. 2022). Notably, compared to the wild‐type plants, the *oswrky53* mutant consistently exhibited a significantly increased tiller number and a reduction in plant height over consecutive years (Tian et al. 2017; Tian et al. 2021; Tang et al. 2022). In this study, we demonstrate that OsWRKY53 negatively regulates tiller formation by directly activating *OsTB1* transcription, and also participates in SL signalling to control rice tiller development. Therefore, our study uncovers a novel biological function of OsWRKY53 in tiller regulation and offers a strategy for breeding cultivars with optimised plant architecture.

## 2 Results

### 2.1 OsWRKY53 Negatively Regulates Tiller Formation in Rice

Our previous studies have reported that the *oswrky53* mutant exhibits mild dwarfism and a moderately increased tiller number, and that OsWRKY53 functions as a positive regulator of BR signalling to modulate plant height (Tian et al. 2017; Tian et al. 2021; Tang et al. 2022). To elucidate the role of OsWRKY53 in shoot branching, we conducted a systematic quantification of tiller numbers in wild‐type (LJ11) and *oswrky53* mutant plants. Comparative analysis demonstrated a substantial increase in tiller number for *oswrky53‐1* (48.3%) and *oswrky53‐2* (47.7%) mutants relative to LJ11 plants (Figures S1 and 1A,B). To further validate this phenotype, we created loss‐of‐function alleles for *OsWRKY53* in the DHX2, LD16, SJ18, and ZKF5 backgrounds by genome editing, which we named *oswrky53*‐DHX2, *oswrky53*‐LD16, *oswrky53*‐SJ18, and *oswrky53*‐ZKF5 (or *w53*‐DHX2, *w53*‐LD16, *w53*‐SJ18, and *w53*‐ZKF5 for simplicity). Consistent with the initial observations, all mutant lines exhibited a significant increase in tiller number compared to their respective wild‐type plants (Figures S2 and S3A–H; Tang et al. 2022). Additionally, plant height was significantly reduced across all *oswrky53* mutant backgrounds (Figure S3I–L). Collectively, these results demonstrate that OsWRKY53 is a key regulator of plant architecture, influencing both plant height and tiller number.

**FIGURE 1:**
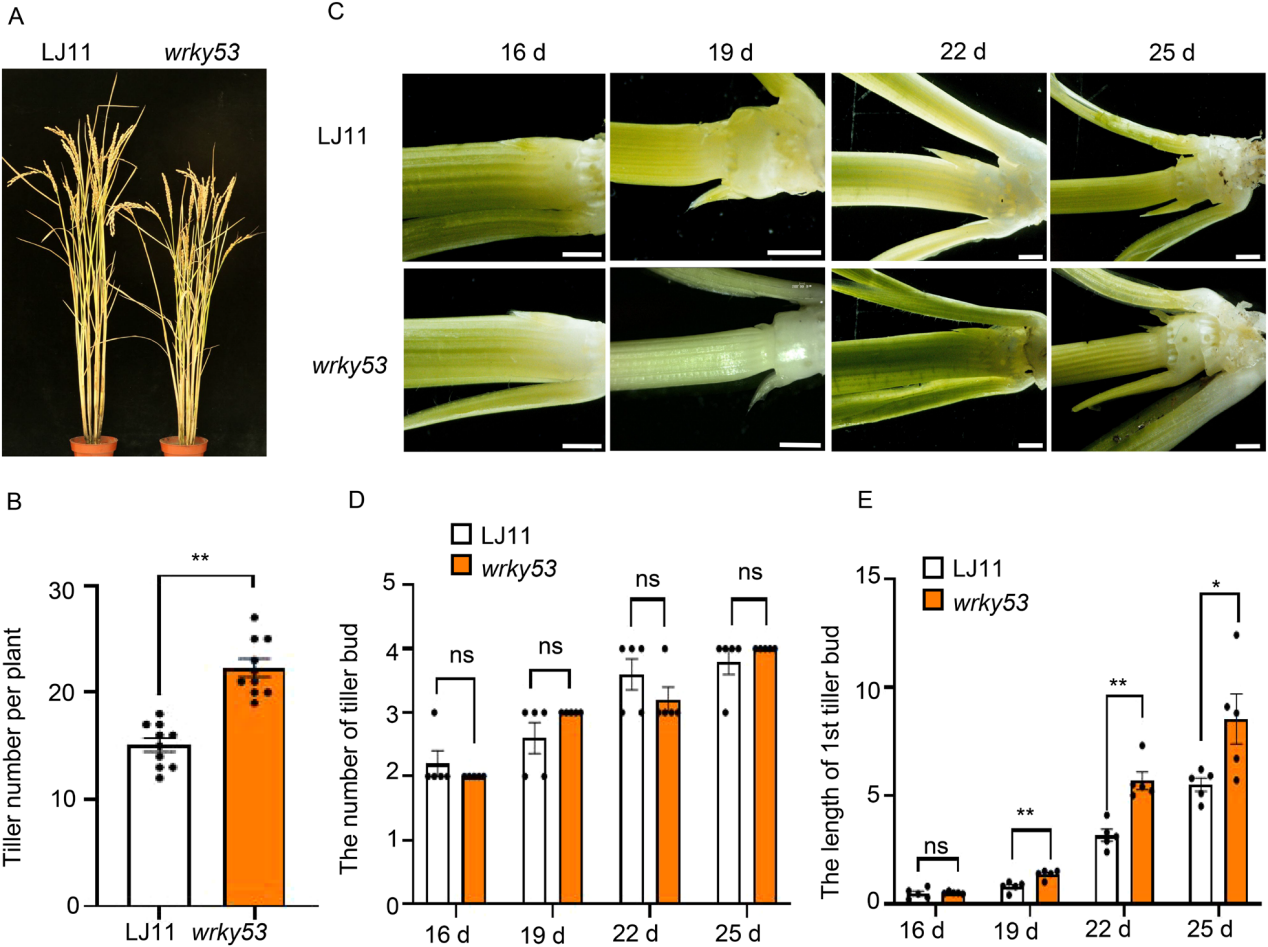
OsWRKY53 negatively regulates tiller formation in rice. (A) Images of plants of LJ11, *oswrky53*. (B) Tiller number per plant of LJ11, *oswrky53*. Data are shown as means ± SE (*n* = 10). (C) Morphologies of shoot basal regions of LJ11 and *oswrky53* in 25‐day‐old plants. Scale bars correspond to 1 mm. (D, E) Number of the tiller bud (D), length of the first tiller bud (E) of LJ11 and *oswrky53* in 25‐day‐old plants. Data are shown as means ± SE (*n* = 5). Each dot represents the result from one biological replicate, error bars indicate means ± SE. *P* values were calculated by Student's *t*‐test, ** is *p* < 0.01, * is *p* < 0.05. ns indicates no significant difference.

The development of tiller buds in rice involves two main processes: the initiation of an axillary bud at each leaf axil and their subsequent outgrowth (Li et al. 2003; Wang and Li 2006; Wang et al. 2018). To dissect the role of OsWRKY53 in this process, we performed a dynamic analysis of tiller bud development at the seedling stage. We observed that axillary bud initiation remained unaffected in *oswrky53* mutants (Figure 1C,D). However, tiller bud growth was significantly accelerated in 19‐day‐old *oswrky53* mutants (Figure 1C,E). These results indicate that OsWRKY53 negatively controls tillering growth to regulate tiller number.

### 2.2 OsWRKY53 Binds to the Promoter of *OsTB1* and Regulates Its Transcription

**FIGURE 2:**
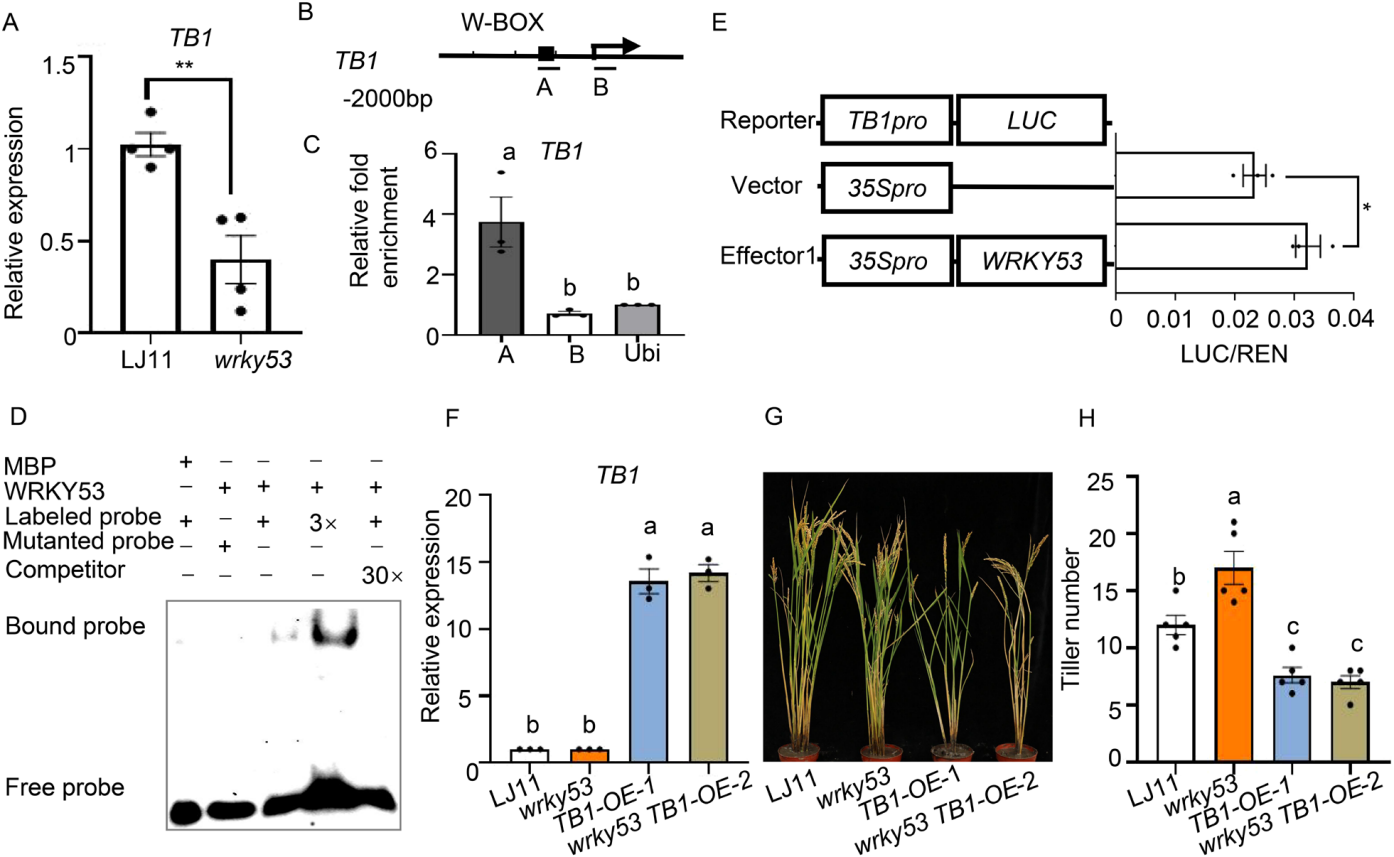
OsWRKY53 binds to the promoters of *OsTB1* and activates their expression. (A) The expression of *OsTB1* in the shoot base of LJ11 and *oswrky53* plants. Data are shown as means ± SE (*n* = 4). (B) Schematic diagrams of the *OsTB1* promoters. The arrows indicate the position of the translation start codon. The scale is 500 bp. DNA fragments (A and B) were used for ChIP and the black squares indicate the W‐box regions (T)TGACC. (C) ChIP assays showing that OsWRKY53 binds to the promoters of *OsTB1* in vivo. Immunoprecipitation was performed with anti‐Flag antibody. Immunoprecipitated chromatin was analysed by RT‐qPCR using primers indicated in (B). RT‐qPCR enrichment was calculated by normalising to ACTIN and to the total input of each sample. Data are shown as means ± SE (*n* = 3). (D) EMSA showing that OsWRKY53 binds to the conserved W‐box of *OsTB1* promoters. A 52‐bp W‐box containing DNA fragment in A region in (B) was used as probe. Versions of the probe with the W‐box mutated to AAAAAA served as mutant probes; unlabeled probes served as competitive probes. MBP was used as a negative control. (E) Schematic diagrams of the effector and reporter plasmids used in the transient assay in rice protoplasts. Transient assay in rice protoplasts showing that OsWRKY53 activates the expression of *OsTB1*. Schematic diagrams of the effector and reporter plasmids are indicated. Reporter together with control or *35Spro:OsWRKY53* effector constructs were transfected into rice protoplasts. Relative LUC activity was calculated as the ratio between firefly luciferase and Renilla luciferase. Data are shown as means ± SE (*n* = 3). Each dot represents the result from one biological replicate; error bars indicate means ± SE. *P* values were calculated by Student's *t*‐test; * is *p* < 0.05. (F) *OsTB1* expression in LJ11, *oswrky53*, *OsTB1‐OE‐1*, *oswrky53 OsTB1‐OE‐2* plants. Data are shown as means ± SE (*n* = 3). (G, H) Representative images of plants (G), and tiller number (H) of LJ11, *oswrky53*, *OsTB1‐OE‐1*, *oswrky53 OsTB1‐OE‐2* mutants at the mature stage. Data are shown as means ± SE (*n* = 5). Each dot represents the result from one biological replicate; error bars indicate means±SE. Statistically significant differences are indicated by different lowercase letters (*p* < 0.05, one‐way ANOVA with Tukey's significant difference test).

To identify the underlying mechanism regulated by OsWRKY53 in rice tillering, we first investigated whether OsWRKY53 directly regulates the expression of key genes involved in tiller development. The expression of *OsTB1* and *OsIPA1* was significantly decreased in *oswrky53* mutants, while the expression of *OsMOC1*, *OsMOC3*, *OsFON1*, *OsSLR1*, *OsGT1* in *oswrky53* mutants remained similar to that in the WT (Figures 2A and S4). These results suggest *OsTB1* may be a potential target gene of OsWRKY53. WRKY‐type transcription factors typically bind to the W‐box (C/TTGACC/T) in the promoters of their target genes. Sequence analysis revealed the presence of the W‐box elements in the promoter regions of *OsTB1* (Figure 2B). ChIP‐qPCR assays further showed that OsWRKY53 binds to the promoter of *OsTB1* in vivo (Figure 2B,C). Electrophoretic mobility shift assay (EMSA) results demonstrated that OsWRKY53 efficiently binds to the promoter regions of *OsTB1* containing the W‐box (Figure 2D). To functionally assess this regulation, we also employed transient expression assays in rice protoplasts using a construct overexpressing *OsWRKY53* (*35Spro:OsWRKY53*) as an effector and the firefly luciferase gene (*LUC*) driven by the promoters of *OsTB1* as reporters. We observed that OsWRKY53 significantly enhanced the expression of the *OsTB1pro:LUC* reporter (Figure 2E). Together, these results indicated that OsWRKY53 can directly bind to the *OsTB1* promoter and activate its expression.

To investigate the genetic relationship between OsWRKY53 and OsTB1 in controlling rice tillering, we next generated *OsTB1*‐overexpression lines in both WT (LJ11) and *oswrky53* mutant backgrounds. Consistent with previous reports (Takeda et al. 2003), *OsTB1‐OE* lines showed significantly reduced tiller numbers (Figure S5), confirming OsTB1's role as a negative regulator of tillering. Then, we selected *OsTB1–OE‐1* and *oswrky53 OsTB1‐OE‐2* lines with comparable transcript levels of *OsTB1* for phenotypic analysis (Figure 2F). Notably, the elevated tiller phenotype characteristic of *oswrky53* mutants was completely suppressed in *oswrky53 OsTB1‐OE‐2* plants, which exhibited tiller numbers similar to *OsTB1‐OE* (Figure 2G,H). These results indicate that OsWRKY53 functions genetically upstream of OsTB1 in the regulation of tiller number.

### 2.3 OsWRKY53‐OsGT1 Cooperative Regulation of OsTB1 in Tiller Control

**FIGURE 3:**
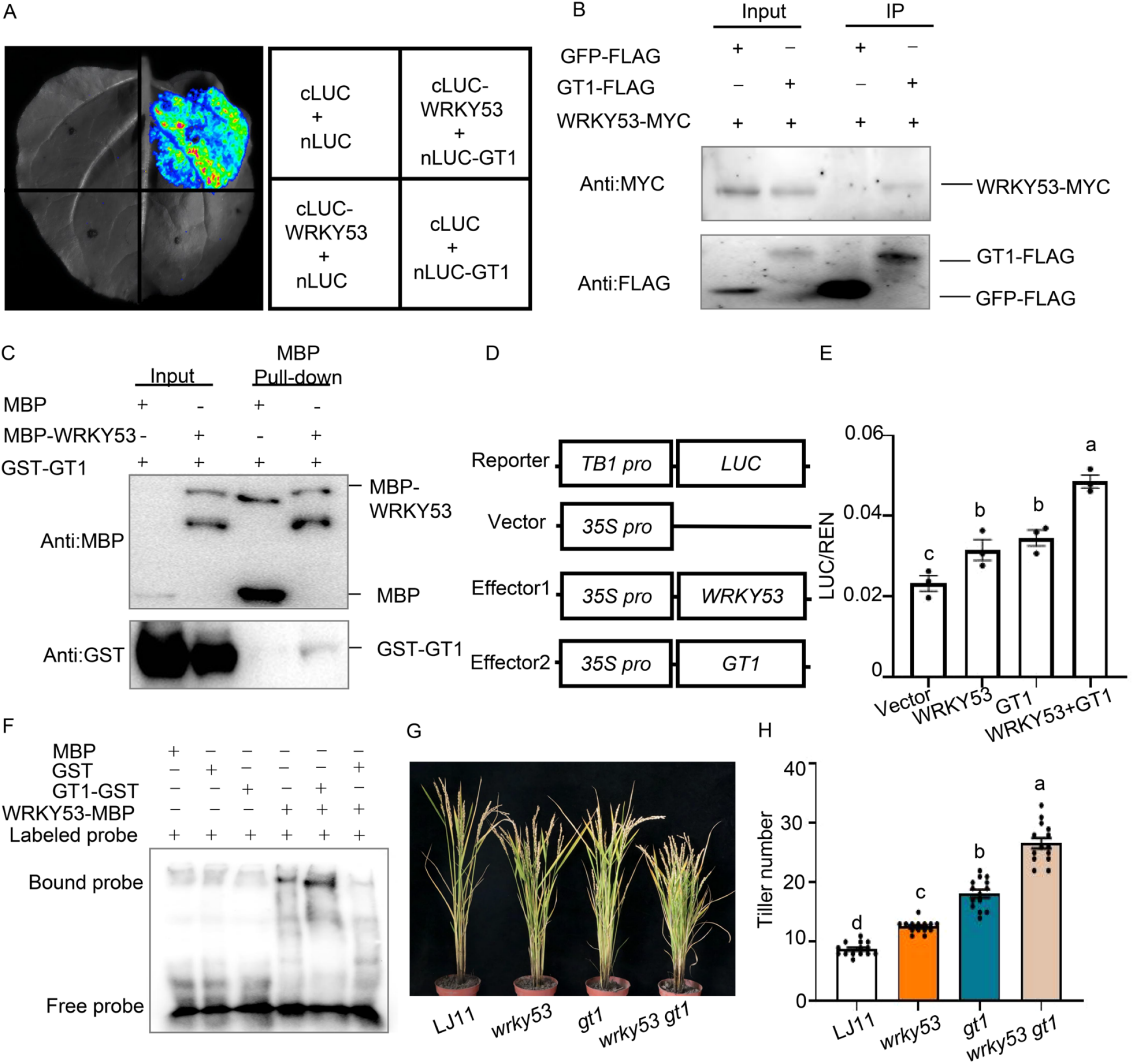
OsGT1 as a co‐activator of OsWRKY53 in regulating tiller formation. (A) LCI assay showing that OsGT1 interacts with OsWRKY53 in *N. benthamiana* leaves. Co‐infiltration of *cLUC‐OsWRKY53* and *nLUC‐OsGT1* constructs leads to the reconstitution of the LUC signal, whereas no signal was detected when *cLUC‐OsWRKY53* and *nLUC*, *cLUC* and *nLUC‐OsGT1*, or *nLUC* and *cLUC* were co‐infiltrated. In each experiment, at least five independent *N. benthamiana* leaves were infiltrated and analysed. (B) The OsWRKY53‐OsGT1 interaction detected by coimmunoprecipitation assays. OsWRKY53‐MYC and OsGT1‐FLAG were transiently co‐expressed in rice protoplasts, and then the immunoprecipitation was performed with the anti‐FLAG antibody. The immunoprecipitated proteins were detected with anti‐FLAG and anti‐MYC antibodies, respectively. (C) Pull‐down assay showing the interaction between OsWRKY53 and OsGT1. GST‐OsGT1 was pulled down by MBP‐OsWRKY53 immobilised on amylose resin beads and was detected with anti‐MBP and anti‐GST antibodies, respectively. (D) Schematic diagrams of the effector and reporter plasmids used in the transient assay in rice protoplasts. (E) Co‐expression of OsGT1 enhances the transcriptional activation of OsWRKY53 on *OsTB1* expression in rice protoplasts. Schematic diagrams of the effector and reporter plasmids are indicated. Reporter together with control, *35Spro:OsWRKY53* or *35Spro:OsGT1* effector constructs were transfected into rice protoplasts. Relative LUC activity was calculated as the ratio between firefly luciferase and Renilla luciferase. Data are shown as means ± SE (*n* = 3). (F) EMSA showing that direct binding of OsWRKY53 to the *OsTB1* promoter is enhanced by the addition of recombinant GST‐OsGT1 protein. GST protein was used as a negative control. (G, H) Representative images of plants (G) and tiller number (H) of LJ11, *oswrky53*, *osgt1*, *oswrky53 osgt1* plants. Data are shown as means ± SE (*n* = 15). Each dot represents the result from one biological replicate; error bars indicate means ± SE. Statistically significant differences are indicated by different lowercase letters (*p* < 0.05, one‐way ANOVA with Tukey's significant difference test).

Previous studies have revealed that OsWRKY53 interacts with OsGT1 in the nucleus (Yang et al. 2022). Given that OsGT1 regulates lateral branching (Whipple et al. 2011), we hypothesized that OsWRKY53 and OsGT1 may function in the same pathway to control rice tillering. First, we performed LCI assay and showed that OsWRKY53 can interact with OsGT1 directly in *Nicotiana benthamiana* leaves (Figure 3A). This OsWRKY53‐OsGT1 interaction was further validated through Co‐IP assay. Co‐expression of OsWRKY53‐MYC and OsGT1‐FLAG in rice protoplasts resulted in successful co‐immunoprecipitation of OsWRKY53‐MYC using the anti‐FLAG antibody (Figures 3B and S6). Additionally, pull‐down assays demonstrated that the GST‐OsGT1 fusion protein can interact with the MBP‐OsWRKY53 fusion protein in vitro (Figures 3C and S7). Together, these results indicate that OsWRKY53 physically interacts with OsGT1 both in vivo and in vitro.

To elucidate the functional relationship between OsWRKY53 and OsGT1 in regulating tiller development, we analysed *OsTB1* expression in the shoot base of *OsGT1‐RNAi* lines (Yang et al. 2022). RT‐qPCR revealed a significant reduction in *OsTB1* transcript levels in *OsGT1‐RNAi* mutants compared to WT plants (Figure S8). Since HD‐ZIP I transcription factors typically recognise pseudopalindromic sequences (CAATNATTG) or consensus binding sites (TAATTA) in their target promoters (Gao et al. 2016). We identified conserved HD‐ZIP I binding motifs (AATNATT) in the *OsTB1* promoter (Figure S9A). EMSA assays confirmed that OsGT1 protein specifically binds to this motif in the *OsTB1* promoter in vitro (Figure S9B). Additionally, dual‐luciferase assays confirmed that OsGT1 activates expression of *OsTB1* (Figure 3D,E), indicating that OsGT1 could directly bind to *OsTB1* promoter and activate its expression. Previous studies have shown that OsTB1/AtTB1 activates *OsGT1* and its orthologs (*AtHB21*, *AtHB40*, *AtHB53*) to suppress tiller formation (González‐Grandío et al. 2017; Kumar et al. 2021). Together with our findings, these results indicate that OsGT1 and OsTB1 form a double‐positive feedback loop, which may establish bistable switches to facilitate rapid developmental responses.

To investigate the mechanisms by which OsWRKY53‐OsGT1 regulates *OsTB1*, we examined whether OsGT1 influences the DNA‐binding activity of OsWRKY53, or conversely, OsWRKY53 modulates that of OsGT1. EMSAs revealed that the DNA‐binding capacity of OsWRKY53 was markedly enhanced in the presence of OsGT1, and similarly, OsGT1 binding was significantly strengthened by OsWRKY53 (Figures 3F, S9C and S10). Transient expression assays demonstrated that co‐expression of OsGT1 and OsWRKY53 significantly enhanced *OsTB1* promoter activity compared to either protein alone (Figure 3D,E). Genetic analysis demonstrated that *osgt1* single mutants developed significantly more tillers than WT controls, while the *oswrky53 osgt1* double mutant exhibited an additive tillering phenotype, exceeding that observed in either the *oswrky53* or *osgt1* single mutants (Figures 3G,H and S11). Together, these results suggest that OsWRKY53 and OsGT1 cooperatively inhibit tiller development through their synergistic control of *OsTB1* expression.

### 2.4 The *oswrky53* Mutation Confers Reduced Sensitivity to SL

**FIGURE 4:**
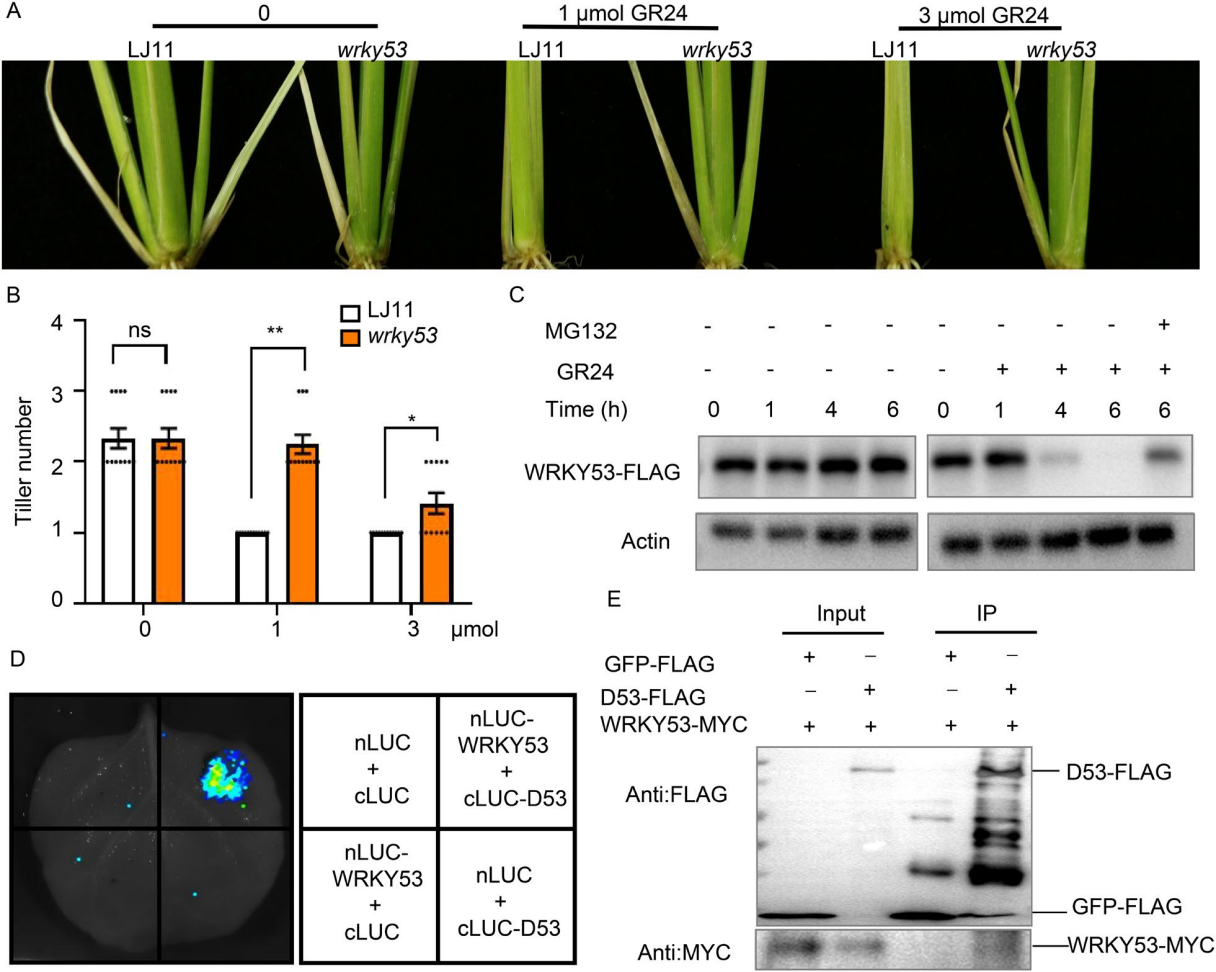
OsWRKY53 physically interacts with D53. (A) Gross morphologies of LJ11, *oswrky53* seedlings with or without rac‐GR24 treatment. Seedlings were treated with 1 μM rac‐GR24, 3 μM rac‐GR24 or mock (0 μM rac‐GR24). (B) Statistical analysis of tiller number in (A). Data are shown as means ± SE (*n* = 12). (C) Time course of degradation of OsWRKY53 protein in the shoot base of 14‐day‐old *OsWRKY53‐FLAG‐OE* seedlings treated with or without 5 μM rac‐GR24. OsWRKY53 level was examined by protein gel blot with anti‐FLAG antibody. ACTIN contents detected with anti‐ACTIN antibodies were used as loading control. (D) LCI assay showing that D53 interacts with OsWRKY53 in *N. benthamiana* leaves. Co‐infiltration of nLUC‐OsWRKY53 and cLUC‐D53 constructs leads to the reconstitution of the LUC signal, whereas no signal was detected when nLUC‐OsWRKY53 and cLUC, nLUC and cLUC‐D53, or nLUC and cLUC were co‐infiltrated. In each experiment, at least five independent *N. benthamiana* leaves were infiltrated and analysed. (E) The OsWRKY53‐D53 interaction detected by coimmunoprecipitation assays. OsWRKY53‐MYC and D53‐FLAG were transiently co‐expressed in rice protoplasts, and then the immunoprecipitation was performed with the anti‐FLAG antibody. The immunoprecipitated proteins were detected with anti‐FLAG and anti‐MYC antibodies, respectively.

OsWRKY53 is a key regulator of BR signalling in rice, with *oswrky53* mutants displaying characteristic BR‐related phenotypes such as erect leaves, decreased plant height, and reduced grain size (Tian et al. 2017; Tian et al. 2021). However, the increased tillering phenotype in *oswrky53* mutants contrasts with typical BR signalling mutants (Fang et al. 2020), implying that OsWRKY53 may participate in other signalling pathways to control rice development. Given the conserved function of SL in regulating plant branching architecture (Gomez‐Roldan et al. 2008; Umehara et al. 2008; Ruyter‐Spira et al. 2011; Rasmussen et al. 2012), we hypothesized that OsWRKY53 might function in SL biosynthesis or signalling. To investigate this possibility, we examined the SL sensitivity of *oswrky*53 mutants in response to different concentrations of rac‐GR24, a synthetic SL analogue. While exogenous rac‐GR24 treatment strongly suppressed tillering in LJ11 plants, *oswrky53* mutants exhibited markedly attenuated responses, maintaining normal tiller numbers at 1 μM GR24 and showing a slight decrease under 3 μM rac‐GR24 (Figure 4A,B). These results indicate that *oswrky53* mutants display significantly compromised SL sensitivity, demonstrating that OsWRKY53 plays a crucial role in the SL signalling pathway.

### 2.5 D53 Interacts With and Promotes the Stability of OsWRKY53

To elucidate the role of OsWRKY53 in the SL signalling pathway, we examined whether SL regulates OsWRKY53 expression. We found GR24 treatment significantly reduced *OsWRKY53* mRNA accumulation (Figure S12). At the protein level, OsWRKY53 also showed marked degradation following 5 μM GR24 treatment (Figures 4C and S13). Notably, the proteasome inhibitor MG132 effectively blocked GR24‐induced OsWRKY53 degradation, suggesting that SL signalling triggers OsWRKY53 protein turnover via the 26 s proteasome pathway (Figures 4C and S13C,D).

Given that D3/MAX2 serves as a key component in SL signalling and that OsBZR1 acts as a substrate for D3 in rice SL signalling (Fang et al. 2020), we investigated possible interactions between OsWRKY53 and D3. However, neither LCI nor yeast two‐hybrid assays detected a physical interaction between OsWRKY53 and D3 (Figure S14). D53, a negative regulator in the SL signalling pathway, which is degraded by the SL‐mediated ubiquitination and proteasomal degradation through the D14‐SCF^D3^ complex (Jiang et al. 2013; Zhou et al. 2013). We hypothesized that D53 might regulate OsWRKY53 protein stability. To test this, we first investigated whether D53 physically interacts with OsWRKY53. LCI assays revealed an interaction of OsWRKY53 with D53 in *Nicotiana benthamiana* leaves (Figure 4D), which was further confirmed by co‐immunoprecipitation (Co‐IP) assays in vivo (Figures 4E and S15), providing strong evidence for a functional association between D53 and OsWRKY53.

**FIGURE 5:**
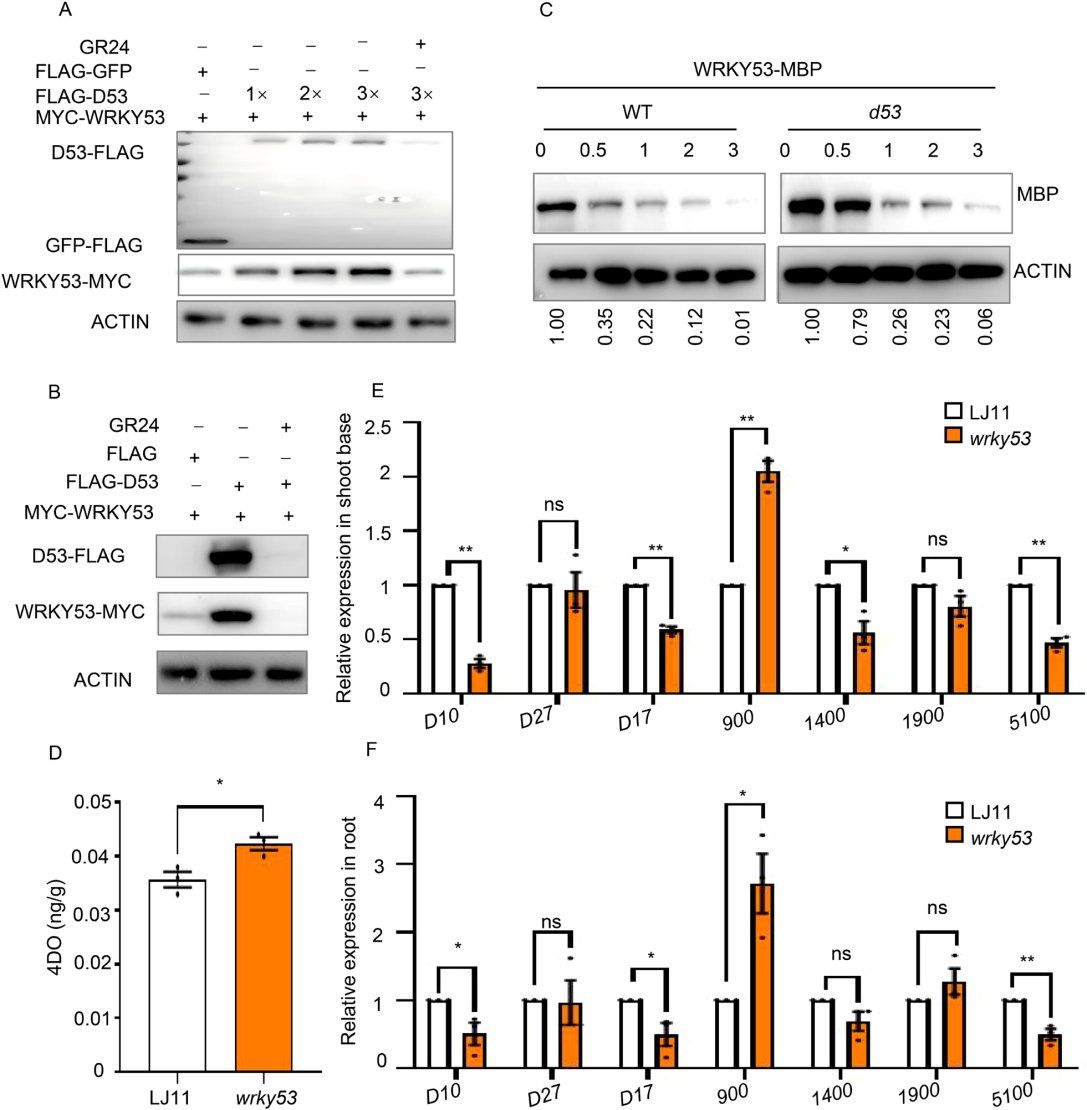
D53 enhances the stabilization of OsWRKY53. D53 promotes the stabilization of OsWRKY53 protein. *35Spro:FLAG‐GFP*, *35Spro:FLAG‐D53*, and *35Spro:MYC‐WRKY53* were transiently expressed in rice protoplast. OsWRKY53 was detected using the anti‐MYC antibody. The loading control of GFP, D53 was detected using the anti‐FLAG antibody. (B) Influence of D53 on OsWRKY53 stability in *N. benthamiana*. OsWRKY53‐MYC was transiently co‐expressed with D53‐FLAG or free FLAG in *N. benthamiana* leaves. OsWRKY53 was detected using the anti‐MYC antibody. The loading control of GFP, and D53 was detected using the anti‐FLAG antibody. (C) In vitro cell‐free degradation assay showing that D53 stabilises OsWRKY53. Total protein extracted from 14‐old‐day Norlin8, *d53* seedlings were incubated with recombinant MBP‐OsWRKY53 protein in the presence of 1 mM ATP. MBP‐OsWRKY53 was detected with anti‐MBP antibody with ACTIN as a loading control. Values below the blots represent the ratio of OsWRKY53‐MBP to ACTIN signals, with that in the first lane arbitrarily set to 1 for comparison. (D) Contents of 4DO in root exudates of LJ11 and *oswrky53* seedlings. The expression of SL biosynthesis genes *D10*, *D27*, *D17*, *Os900*, *Os1400*, *Os1900*, and *Os5100* in shoot base of LJ11 and *oswrky53* plants. (F) The expression of SL biosynthesis genes *D10*, *D27*, *D17*, *Os900*, *Os1400*, *Os1900*, and *Os5100* in the roots of LJ11 and *oswrky53* plants. Data are shown as means ± SE (*n* = 3). Each dot represents the result from one biological replicate; error bars indicate means ± SE. *P* values were calculated by Student's *t*‐test, ** is *p* < 0.01, * is *p* < 0.05. ns indicates no significant difference.

To investigate the biochemical effect of D53 on OsWRKY53 protein stability, we co‐expressed MYC‐OsWRKY53 with D53‐FLAG or a GFP‐FLAG control in rice protoplasts. Co‐expression of D53‐FLAG led to a dose‐dependent accumulation of OsWRKY53 protein (Figures 5A and S16). Notably, GR24 treatment induced D53 degradation and simultaneously reduced the abundance of OsWRKY53 (Figures 5A and S17), thereby connecting SL signalling to this regulatory module. Similarly, in *N. benthamiana* leaves, co‐expression of OsWRKY53‐MYC with D53‐FLAG increased OsWRKY53 protein levels, an effect that was abolished by GR24 treatment (Figures 5B and S18). To further elucidate the role of D53 in stabilising OsWRKY53, we performed a cell‐free degradation assay using the multi‐branch *d53* mutant, which carries a gain‐of‐function mutation and is insensitive to SL treatment (Zhou et al. 2013). In this assay, MBP‐OsWRKY53 was incubated with protein extracts from either the *d53* mutant or WT (Norin 8) plants. The results showed that MBP‐OsWRKY53 degradation was significantly slower in *d53* mutant extracts than in WT extracts (Figures 5C and S19). These results indicate that D53 physically interacts with and stabilises OsWRKY53, and that SL‐induced degradation of D53 releases this stabilisation, leading to OsWRKY53 turnover. Collectively, our findings support a model in which the D53‐OsWRKY53 regulatory module operates within the SL signalling pathway to fine‐tune tiller development in rice.

### 2.6 OsWRKY53 Modulates SL Biosynthesis Gene Expression in Rice

Previously reported SL signalling mutants, such as *d53* and *d14*, accumulate elevated levels of SLs due to disrupted negative feedback regulation. For instance, 4‐deoxyorobanchol (4DO) content in these mutants has been reported to be approximately 30% or 4‐fold higher than in the wild type (Jiang et al. 2013; Abe et al. 2014; Seto et al. 2014; Hu et al. 2024). To investigate whether OsWRKY53 influences SL homeostasis, we measured SL content in root exudates. The *oswrky53* mutants showed a slight but reproducible increase in 4DO levels (~18%) compared with the wild type (Figure 5D), suggesting that OsWRKY53 acts as a weak attenuator within the SL signalling pathway. We next examined the expression of key SL biosynthetic genes including *D10*, *D17*, *D27*, *Os900*, *Os1400*, *Os1900*, *Os5100* in shoot base and root. Quantitative analysis revealed that in both shoot bases and root, *D10, D17*, and *Os5100* were significantly down‐regulated in *oswrky53* mutants, whereas *Os900* was markedly up‐regulated (Figure 5E,F). In the SL biosynthetic pathway, *D27*, *D17*, and *D10* sequentially convert all‐trans‐β‐carotene to carlactone (CL). Subsequently, *Os900*, *Os1400*, *Os1900*, and *Os5100* convert CL into carlactonoic acid (CLA). CLA is then transformed into 4DO primarily by *Os900*, and 4DO is further modified to orobanchol by *Os1400* (Yoneyama et al. 2018). We explain that up‐regulation of *Os900* in *oswrky53* mutants mainly accounts for the increase in SL content. Using the unpublished Rice Regulome Atlas database (http://plantencode.fun/regulome/rice/), we analysed the binding profile of OsWRKY53 to SLrelated biosynthetic genes. Available‐ ChIP‐seq data indicated binding of OsWRKY53 to the promoter of *D10*, and both ChIP‐seq and DAP‐seq datasets showed strong binding peaks at *Os5100*. No significant peaks were detected at *D17* or other *MAX1* homologues (Figure S20). To validate these predictions, we focused on *D10*. Bioinformatics analysis confirmed the presence of canonical W‐box (C/TTGACC/T) motifs in the *D10* promoter (Figure S21A). ChIP‐qPCR assays in *OsWRKY53‐FLAG‐OE* transgenic plants showed strong enrichment of OsWRKY53 at the P2 region of the *D10* promoter, but not at other tested regions (Figure S21B). EMSAs further demonstrated that OsWRKY53 binds directly to the P2 fragment in vitro (Figure S21C). Taken together, these findings indicate that OsWRKY53 directly or indirectly modulates the transcription of key SL‐biosynthesis genes (including *D10*, *D17*, *Os900* and *Os5100*), thereby influencing SL content and contributing to the feedback regulation of SL signal transduction.

### 2.7 Evaluation of the Breeding Potential of the OsWRKY53 and OsGT1

As previously reported, although *oswrky53* plants produced more tillers, they did not show a significant increase in overall yield due to a reduction in 1000‐grain weight (Tang et al. 2022). This prompted us to hypothesize whether knocking out *OsWRKY53* in a large‐grain, high‐quality rice variety could enhance yield by promoting tiller formation. We selected ZKF5, an elite variety known for its high yield and superior grain quality, which possesses elite genotype combining *GL7*, *gs3*, and *GW5*, resulting in long and slender grains (Chen et al. 2023). To test our hypothesis, we analysed the phenotype of *oswrky53*‐ZKF5 and found that the tiller number of *oswrky53*‐ZKF5 increased by approximately 35.16%, whereas its 1000‐grain weight decreased by approximately 19.78% (Figures S3H and S22). Unfortunately, the yield per plant of *oswrky53*‐ZKF5 remained comparable to that of the wild‐type ZKF5 (Figure S22).

**FIGURE 6:**
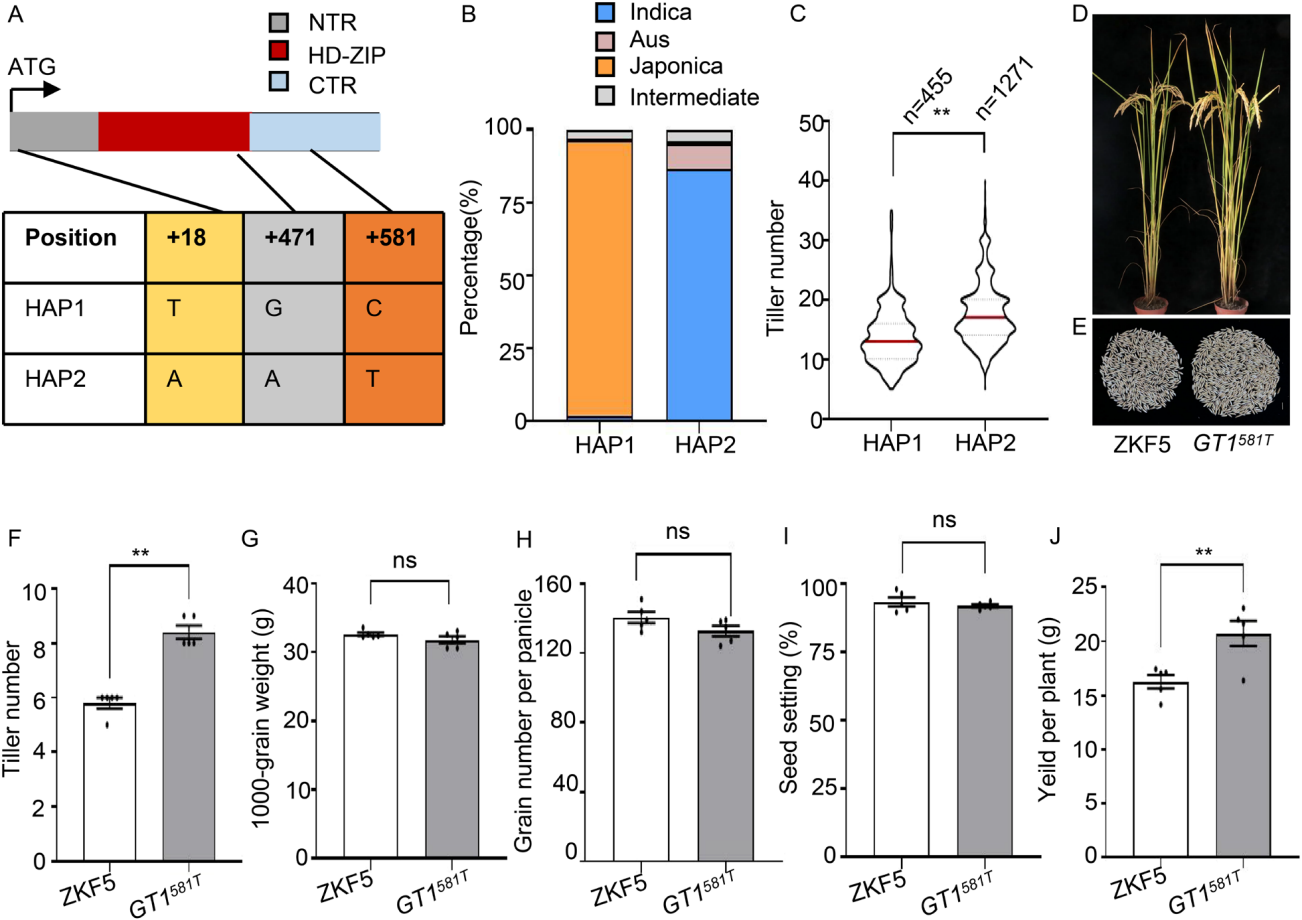
*OsGT1‐581* is a functional variation for regulating tiller formation. (A) Sequence analysis of the *OsGT1* coding region. The position of SNP‐581 is presented. (B) Distribution of the *OsGT1‐581C* and *OsGT1‐581T* polymorphisms among rice ecotypes. (C) Varieties with the HAP2‐*OsGT1* allele exhibit more tillers than varieties with the HAP1‐*OsGT1* allele. (D, E) Representative images (D) and grain yield per plant (E) of ZKF5 and *OsGT1* ^*581T*^(ZKF5). (F–J) Summary of agronomic traits in ZKF5 and *OsGT1* ^*581T*^(ZKF5) plants grown in a paddy field. Data are shown as means ± SE (*n* = 5). Each dot represents the result from one biological replicate. *P* values were calculated by Student's *t* test. ns indicates no significant difference. ** is *p* < 0.01.

Next, to evaluate the breeding potential of OsGT1, we analysed its coding sequences in 4722 diverse rice accessions from RiceVarMap v2.0 (Zhao et al. 2015). This analysis identified three SNPs that define two major haplotypes: HAP1 (predominant in *japonica*, 94.7%) and HAP2 (predominant in *indica*, 86.7%; Figure 6A,B). Phenotypic association analysis using culm number data from 1726 accessions demonstrated that accessions carrying HAP2 produced significantly more tillers than those with HAP1 (Figure 6C; Table S1), suggesting OsGT1 variation contributes to tiller number regulation. Among these three SNPs, SNP‐471G/A is synonymous, whereas SNP‐18T/A and SNP‐581C/T lead to amino acid substitutions (Asp6Glu and Ala194Val, respectively; Figure 6A). Bioinformatics prediction (ricevarmap.ncpgr.cn) indicated both nonsynonymous SNPs likely affect OsGT1 function, with SNP‐581 showing a greater potential impact based on chromatin accessibility metrics (Figure S23).

To directly evaluate the functional significance of *OsGT1*‐SNP‐581 C/T in determining tiller number, we created *OsGT1* ^*581T*^ plants in ZKF5 background by *ePE2* prime editors (Figure S24). The *OsGT1* ^*581T*^(ZKF5) plants exhibited a significantly higher tiller number compared to ZKF5 controls (Figure 6D,F). However, no significant differences were observed in grain number per panicle, seed setting rate, or 1000‐grain weight. Notably, the yield per plant increased by 27.7% (Figure 6E–J). Together, these findings suggest that natural variation in *OsGT1*, particularly SNP‐581, is functionally involved in controlling rice tiller formation. The *OsGT1* ^*581T*^ allele (HAP2, *indica*) likely represents a superior allele for enhancing tiller number and improving yield in *japonica* rice breeding.

## 3 Discussion

Tiller number and plant height, as key determinants of plant architecture, significantly influence yield formation (Wang and Li 2006). The widespread adoption of semi‐dwarf varieties enabled by the *sd1* gene represented a milestone in rice breeding and played a pivotal role in the Green Revolution. However, *sd1* remains the only semi‐dwarf gene utilised in stem height control within GR varieties, which significantly limits the development of novel elite cultivars. Given the ongoing rapid growth of the global population, there is an increasing need to raise total grain output. The semi‐dwarf, multi‐branching mutant *oswrky53* exhibits enhanced resistance to sheath blight (Yuan et al. 2020; Gao et al. 2021; Yang et al. 2022), bacterial blight (Xie et al. 2021), and the striped stem borer (Hu et al. 2016), along with higher cold tolerance at the booting stage (Tang et al. 2022), and improved salt tolerance (Yu et al. 2023). These attributes underscore the considerable potential of *oswrky53* in breeding programs aimed at developing high‐yielding, stress‐resilient crop varieties.

SLs play pivotal roles in regulating shoot branching and root architecture (Gomez‐Roldan et al. 2008; Umehara et al. 2008; Ruyter‐Spira et al. 2011; Rasmussen et al. 2012). Our study reveals that *oswrky53* mutants exhibit reduced sensitivity to SL treatment, supporting the role of OsWRKY53 in mediated SL signalling. Furthermore, we identified a physical interaction between D53 and OsWRKY53 (Figure 4D,E) and found that D53 stabilises the protein level of OsWRKY53 (Figure 5A–D). Notably, OsWRKY53 does not interact with D3, indicating that its stabilisation by D53 is independent of D3 (Figure S14). This observation implies that additional E3 ligases may be involved in regulating OsWRKY53 protein levels, warranting further investigation. Previous studies have shown that D53 interacts with OsIPA1 to repress its transcriptional activity, while OsIPA1 directly activates *D53* expression, establishing a feedback loop for SL‐induced *D53* expression (Song et al. 2017). This prompted us to investigate whether OsWRKY53 regulates *D53* transcription. Although several W‐box motifs are present in the *D53* promoter, ChIP‐qPCR assays revealed no direct binding of OsWRKY53 to this region (Figure S25A,B). Consistently, transcriptional analysis showed no difference in *D53* expression between WT and *oswrky53* mutants. Dual‐luciferase assays further confirmed that OsWRKY53 does not influence the transcriptional activity of the *D53* promoter (Figure S25C,D).

**FIGURE 7:**
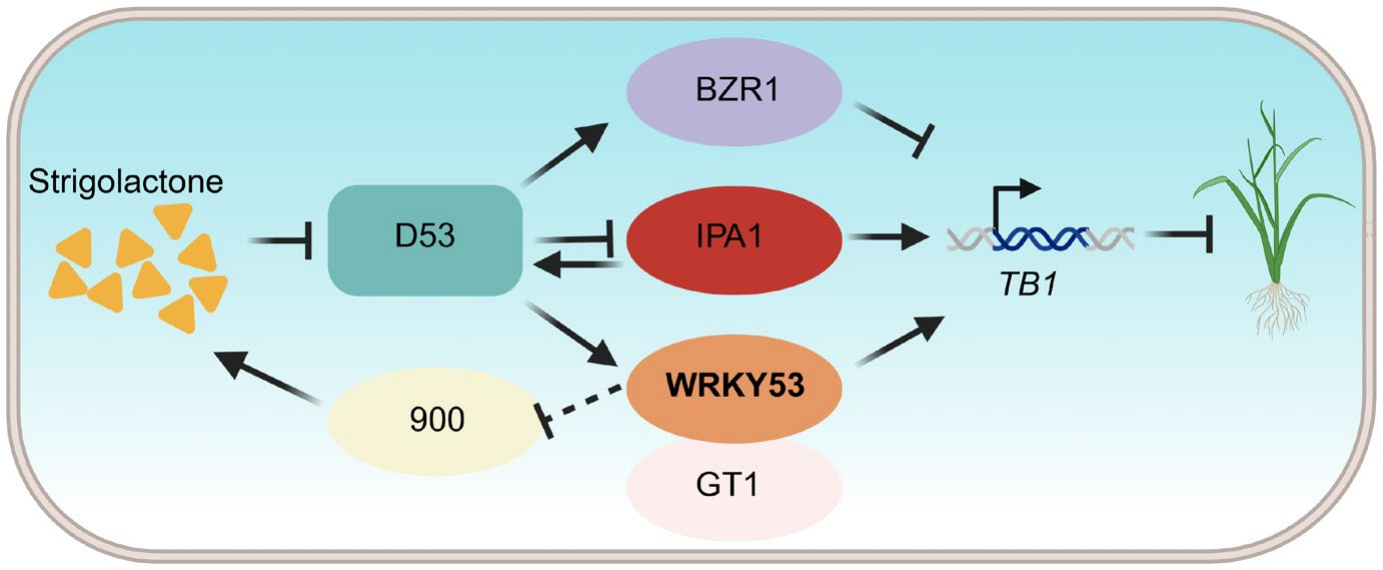
Working model of OsWRKY53 in regulating rice tiller development and fine‐tuning SL signalling. OsWRKY53 inhibits rice tillering by interacting with OsGT1 to co‐activate *OsTB1* transcription. In SL signalling pathway, D53 oppositely regulates expression of *OsTB1* through different partners. D53 can relieve OsIPA1‐mediated activation of *OsTB1* expression and enhance OsBZR1‐dependent repression of *OsTB1*. OsIPA1 and OsBZR1 act as primary partners, whereas OsWRKY53 serves as a minor partner. OsWRKY53 is stabilised by D53 and contributes to *OsTB1* activation, establishing a buffering mechanism that balances tillering and yield. Additionally, as a regulatory component of SL signalling, OsWRKY53 negatively modulates SL biosynthesis, further fine‐tuning the pathway. Created with BioRender (https://BioRender.com/woufypt).

D53 is a critical negative regulator of SL signalling, which functions in SL‐mediated tiller development via repressing *OsTB1* expression (Fang et al. 2020). Previous studies have shown that D53 can relieve OsIPA1‐mediated activation of *OsTB1* expression (Song et al. 2017), and enhance OsBZR1‐dependent repression of *OsTB1* (Fang et al. 2020). In this study, we found D53 interacts with and stabilises OsWRKY53 (Figures 4D,E, 5A–C and S15–S19), and OsWRKY53 activates expression of *OsTB1* (Figure 2), which is inconsistent with the established role of D53 in repressing *OsTB1* and promoting tiller number. We propose a model in which D53 oppositely regulates expression of *OsTB1* through different partners (Figure 7). OsIPA1 and OsBZR1 act as primary partners, ensuring the repressive effect of D53 on *OsTB1*, whereas OsWRKY53 serves as a minor partner that modulates this repression, providing a buffering mechanism. This multiple layer of regulation of *OsTB1* by D53 may help fine‐tune tiller development, preventing both excessive and insufficient branching, thereby supporting balanced growth and optimal yield formation in rice (Figure 7).

Interestingly, SL directly regulates sugar allocation to control tillering, whereas the OsWRKY53‐OsGT1 complex activates *OsSWEET2a/3a*, hexose transporters linked to sheath blight resistance (Feng et al. 2025; Yang et al. 2022). ABA accumulation in shoot buds correlates with branching, and SL signalling induces OsGT1/AtHB40 to promote ABA biosynthesis (Gonzalez‐Grandio et al. 2017; Liu et al. 2020). It will be worthwhile to investigate whether OsWRKY53 is involved in these pathways.

In our current and previous studies, we observed that although *oswrky53* plants produced more tillers, they did not show a significant increase in overall yield due to a reduction in 1000‐grain weight (Figure S26; Tang et al. 2022). Similarly, *oswrky53 osgt1* plants also exhibited a markedly higher tiller number, but this was accompanied by a significant decrease in both grains per panicle and 1000‐grain weight. Consequently, the yield per plant remained comparable to that of the wild type (Figure S26). Balancing the trade‐off relationships among yield components, such as tiller number, grains per panicle, 1000‐grain weight, and seed‐setting rate, remains a central challenge in breeding programs. The well‐characterised OsIPA1 provides a relevant precedent. A single mutation in Os*IPA1* disrupts the miRNA‐directed regulation by *OsmiR156*, leading to an ideal plant architecture with reduced tillering, enhanced lodging resistance, increased grains per panicle, higher grain weight, and ultimately greater yield. These results demonstrate that OsIPA1 is a pleiotropic regulator controlling multiple agronomic traits (Jiao et al. 2010). Similarly, OsWRKY53 exhibits pleiotropic effects, with *oswrky53* mutants displaying a semi‐dwarf phenotype, increased tillering, and compact plant architecture—traits associated with enhanced lodging resistance. Additionally, these mutants show improved tolerance to cold and salt stress, stronger disease resistance, and delayed senescence. Given these multifaceted roles, it would be promising to pyramid the *oswrky53* allele with other favourable grain‐size regulators that act independently of BR signalling, such as *GW5*, *GW2*, *GS3*, and *GL7* (Duan et al. 2017; Fan et al. 2006; Song et al. 2007; Wang et al. 2015). This pyramiding breeding could overcome the small‐grain defect of *oswrky53* while providing a viable strategy for improving plant architecture, enhancing yield, and boosting stress resistance. Additionally, we found that varieties containing the *OsGT1* ^*581T*^ allele showed a significantly increased tiller number, without introducing other undesirable traits, which contributed to higher yield (Figure 6D–J). Taken together, these findings demonstrate that both the *oswrky53* mutant and the *OsGT1* ^*581T*^ allele represent valuable genetic resources for breeding rice varieties with ideal plant architecture and high yield potential in the future.

## 4 Materials and Methods

### 4.1 Plant Materials and Growth Conditions

The rice (*Oryza sativa*) cultivars used in this study are: Longjing11 (LJ11), Daohuaxiang 2 (DHX2), Longdao16 (LD16), Suijing18 (SJ18), Zhongkefa5 (ZKF5) plants were grown in the field (under natural long day conditions) or in a greenhouse at 30°C for 16 h (day) and 24°C for 8 h (night). Rice Dongjing (DJ) plants, *OsGT1‐RNAi* plants (Yang et al. 2022) were grown in the greenhouse at 30°C for 10 h (day) and 24°C for 14 h (night). The agronomical traits, including plant height, effective tiller number, grain number per panicle, 1000‐grain weight, seed setting rate and yield per plant, were measured. Tiller buds were observed directly under a microscope (Olympus SZX16).

### 4.2 Generation of Transgenic Rice Plants

The *oswrky53* mutants in the Longjing11, Daohuaxiang2, and Longdao16 backgrounds were described previously (Tang et al. 2022). In this study, we used *oswrky53‐2*, *oswrky53‐1*, *w53‐*DHX2, and *w53*‐LD16 for analysis; *oswrky53‐2* is used for most of the experiments and named as *wrky53* if no indicated. To generate loss of function *oswrky53* mutants in the Suijing18 (SJ18) and Zhongkefa5 (ZKF5) cultivars, we used previously published gene editing vectors targeting *OsWRKY53* for transformation (Tian et al. 2017). For the generation of the *osgt1* mutants in the Longjing11 and *oswrky53* backgrounds, target sequences for *OsGT1* (Table S2) were synthesised, ligated into respective sgRNA constructs, and sequentially assembled into the CRISPR/Cas9 binary vector pYLCRISPR/Cas9P_ubi_‐H as described. To generate the *OsTB1* overexpression plants, the coding regions of *OsTB1* (Os03g0706500) were amplified by PCR and cloned into *p1390U*. For the *OsWRKY53‐FLAG‐OE* plant, the coding regions of *OsWRKY53* (Os05g0343400) were amplified and cloned into the *pCambia1300‐FLAG* vector. To generate the *OsGT1* ^*581T*^ mutants in the ZKF5 background, the protospacer sequence, sgRNA scaffold, RTT‐PBS‐linker component, and evopreQ1 for *OsGT1‐581(C/T)* (Table S2) were synthesised, annealed, and assembled into the *ePE2* vector by the Golden Gate cloning method (Li et al. 2023). Mutation sites and expression levels of corresponding genes were verified by DNA sequencing, RT‐qPCR, and western‐blot analysis.

### 4.3 GR24 Application Experiment

Two‐week‐old hydroponically cultured rice seedlings were grown in a climatic cabinet at 80% humidity, under 16 h light at 25°C and 8 h dark at 16°C. The seedlings were treated with 1, 3 μM rac‐GR24 (Solarbio, Lot NO 1114I025) or an equivalent volume of ethanol to observe and quantify tiller number. For molecular analyses, seedlings were treated with 5 μM rac‐GR24 or an equal volume of ethanol, then shoot base segments (0.5 cm) were harvested at indicated time points. RNA isolation, cDNA synthesis, qRT‐PCR, and protein extraction were performed as described above.

### 4.4 Total RNA Isolation and RT‐qPCR Analysis

Total RNA was extracted using TRIzol reagent (Invitrogen) and treated with DNase I. First strand cDNAs were synthesised from 2 μg of total RNA using Superscript II Reverse Transcriptase (Invitrogen). Real‐time PCR was performed with SYBR Green PCR master mix (Takara). Data were collected using a Bio‐Rad chromo 4 real‐time PCR system. Expression values were normalised to *UBIQUITIN* (Os01g0328400). The primers used are listed in Table S2. Three or four biological repeats with three technical repeats per biological repeat were performed for each analysis. Values are means ± SE of three or four biological repeats.

### 4.5 Chromatin Immunoprecipitation (ChIP) Assay

Two‐week‐old *OsWRKY53‐FLAG* overexpression seedlings were used for ChIP assays. Briefly, 2 g of rice plant leaf were vacuum‐infiltrated for 30 min with crosslinking solution containing 1% formaldehyde. Crosslinking was then stopped by the addition of 5 mM glycine for 5 min. Samples were ground into fine powder in liquid nitrogen and used to isolate nuclei. Protein–DNA complexes were immunoprecipitated with anti‐FLAG antibody (M20008L, Abmart). The precipitated DNA was recovered and analysed by quantitative PCR. Chromatin precipitated without antibody served as a negative control. The primers for RT‐qPCR used are listed at Table S2. Data are presented as means ± SE of three biological repeats, each consisting of three technical replicates.

### 4.6 Electrophoretic Mobility Shift Assays (EMSA)

The full‐length coding sequences of *OsWRKY53 and OsGT1* were amplified from *Nipponbare* cDNAs and cloned into the *pVP13* or *pDEST15* vector via LR recombination reaction to generate the *MBP‐OsWRKY53* and *GST‐OsGT1* fusion protein constructs, respectively. These constructs were transformed into *E. coli* strain BL21(DE3) for expressing the fusion proteins. The recombinant proteins were affinity purified using Dextrin Beads 6FF (SMART LIFESCIENCE, Cat No SA026010) and Glutathione Beads (SMART LIFESCIENCE, Cat No SA008050). Oligonucleotide probes of ~52 bp containing the wild‐type W‐box (TGACC) or a mutated W‐box (AAAAA) motif and the wild‐type C(AATNATT)G pseudopalindromic binding site or a mutated C(CCCCCCC)C sequence were synthesised and labelled with biotin using the EMSA Probe Biotin Labeling Kit (Beyotime, Cat No GS008). For unlabeled probe competition, an unlabeled probe was added to the reactions. EMSA was performed using a Chemiluminescent EMSA kit (Beyotime, Cat No GS009). Probe sequences are shown in Table S2.

### 4.7 Transient Transcription Dual‐Luciferase (Dual‐LUC) Assays

The *35Spro:OsWRKY53* and *35Spro:OsGT1* constructs were generated and used as effectors. To generate the *TB1pro:LUC* as reporters, *OsTB1* promoter was cloned into the *pGreenII 0800‐LUC* vector (Table S2). The resulting effector and reporter constructs were co‐transformed into protoplasts. Then, the protoplast cells were subjected to luciferase activity assays. Renilla luciferase (*REN*) driven by the *35S* promoter in the *pGreenII 0800‐LUC* vector was used as an internal control. Firefly LUC and REN activities were measured with a Dual‐Luciferase reporter assay kit using a GloMax 20/20 luminometer (Promega). LUC activity was normalised to REN activity. For each plasmid combination, three independent transformations were performed with three technical repeats per independent transformation. Values are means ± SE of three independent transformations.

### 4.8 Protein Gel Blot Analysis

For the secondary antibody in the protein gel blot assay, peroxidase‐labelled goat anti‐rabbit antibody (Abcam, ab6721) was utilised. Membranes were developed with the Super signal west pico chemiluminescent substrate kit (Pierce Biotechnology) and the signal was detected by chemiluminescence imaging (Tanon 5200).

### 4.9 Yeast Two‐Hybrid Assay

The coding sequence of *D3* was cloned into the *EcoR I* and *Pst I* sites of the *pGBKT7* vector to generate the *BD‐D3* constructs. The coding sequence of *OsWRKY53* was cloned into the *EcoR I* and *Xho I* sites of the *pGADT7* vector to generate the *AD‐OsWRKY53* construct. The resulting constructs were transformed into yeast strain *Y2H* Gold. The presence of the transgenes was confirmed by growth on an SD/Trp‐Leu‐ plate. To assess protein interactions, the transformed yeast cells were suspended in liquid SD/Trp‐Leu‐ to OD600 = 1.0. The suspended cells were spread on plates containing SD/Trp‐Leu‐His‐ and SD/Trp‐Leu‐His‐Ade‐ medium. Interactions were observed after 3 d of incubation at 30°C.

### 4.10 LCI (LUC Complementation Imaging) Assays

The *nLUC‐D3*, *nLUC‐OsGT1*, *nLUC‐WRKY53, cLUC‐OsWRKY53*, and c*LUC‐D53* constructs were generated using primers shown in Table S2. *Agrobacteria* harbouring different combinations of constructs were co‐infiltrated into *N. benthamiana*, and the infiltrated leaves were analysed for LUC activity using Chemiluminescence imaging (Tanon 5200) after 48 h infiltration.

### 4.11 Pull‐Down Assay

The full‐length coding region of *OsGT1* in *pENTR/D‐TOPO* was subcloned into the expression vector *pDEST15* to generate the glutathione S‐transferase *(GST)‐OsGT1* fusion vector. The coding region of *OsWRKY53* was ligated into the *pVP13* vector to generate the *MBP‐OsWRKY53* constructs using primers shown in Table S2. These constructs were transformed into *Escherichia coli* (Strain *BL21*) to express the protein, and the fusion proteins were purified using corresponding affinity chromatography. MBP or MBP‐OsWRKY53 was coupled to MBP beads for 2 h at 4°C and subsequently incubated with GST‐OsGT1 for 2 h, then washed thoroughly, boiled in 1× SDS‐PAGE sample buffer, and analysed by immunoblot using anti‐GST antibody (Abmart, M20007M) and anti‐MBP antibody (CWBIO, CW0288M), respectively.

### 4.12 Co‐Immunoprecipitation Assays

For Co‐IP assays, *35Spro:D53‐FLAG*, *35Spro:OsGT1‐FLAG*, *35Spro:GFP‐FLAG* and *35Spro:MYC‐OsWRKY53* constructs were prepared as described in Table S2. These plasmids were transiently co‐expressed in rice protoplasts in the indicated combinations. Total protein was extracted using the lysis buffer (50 mM Tris–HCl, pH 7.5, 150 mM NaCl, 0.5 mM EDTA, pH 8.0, 10% glycerol, 0.5% Triton X‐100) with freshly added 10 mM PMSF, a protease inhibitor cocktail (Roche, 11 873 580 001) and 20 μM MG132. Extracts were clarified by centrifugation at 4°C for 10 min. For Co‐IP, 20 μL Protein‐A/G magnetic beads (L00277; GenScript) were pre‐incubated with 2 μL anti‐FLAG (M20008L, Abmart) for 2–3 h with gentle rotation at 4°C; subsequently, extractions of equal total proteins were added for incubation for 3 h at 4°C. The beads were washed four times with wash buffer (50 mM Tris–HCl, pH 7.5, 100 mM NaCl, 0.5 mM EDTA, pH 8.0, 0.1% Triton X‐100). Immunoprecipitated proteins were eluted with 1 × SDS sample buffer, separated on 12% SDS‐PAGE, transferred to PVDF membrane (IPVH00010), and detected with anti‐MYC (M20002L, Abmart) and anti‐FLAG (M20008L, Abmart) antibodies, respectively.

### 4.13 OsWRKY53 Stability Assay

*35Spro:D53‐FLAG* and *35Spro:OsWRKY53‐MYC* constructs were co‐transfected into rice protoplasts or infiltrated into *Nicotiana benthamiana* leaves. For protoplast assays, cells co‐expressing *OsWRKY53‐MYC* and *D53‐FLAG* were incubated overnight in the dark at 28°C, then treated with 2 mM GR24 for 6 h to assess SL induced effects on the D53‐OsWRKY53 interaction. For *Nicotiana benthamiana* assays, leaves were injected with 5 μM GR24 after 48 h infiltration and harvested 24 h later for analysis. Total protein was extracted from protoplasts or *N. benthamiana* leaf tissues using SDS buffer (1 mM PMSF, 1 mM PI), followed by the addition of 5× loading buffer and boiling for 5 min. For GR24 treatment time‐course assay, *OsWRKY53‐FLAG* seedlings were treated with or without 5 μmol/L GR24 for different time points, and detected the OsWRKY53 protein using anti‐FLAG (M20008L, Abmart) antibodies and anti‐ACTIN antibody (M20009M, Abmart).

### 4.14 Cell‐Free Degradtion Assays

Protein extracts were prepared from 10‐day‐old seedlings using degradation buffer (25 mM Tris–HCl, pH 7.5, 10 mM NaCl, 10 mM MgCl_2_, 4 mM PMSF, 5 mM DTT, and 10 mM ATP). Each reaction mixture containing the extract was combined with 40 μg of purified recombinant MBP‐OsWRKY53 protein and incubated at 22°C. At the indicated time points, an aliquot was removed and mixed with 5 × SDS loading buffer to terminate the degradation, then boiled for 5 min. Samples were separated by 10% SDS‐PAGE and analysed by immunoblotting with an anti‐MBP antibody (CWBIO, CW0288M).

### 4.15 Quantification of Strigolactones From Rice Root Exudates

Extraction and purification of strigolactones (SLs) from root exudates was performed as described previously (Chu et al. 2017; Hu et al. 2024) with the following modifications. Briefly, the hydroponic culture medium was collected and D3‐4DO was added as an internal standard for quantification. The medium was loaded onto a pre‐conditioned Oasis HLB cartridge (Waters) for purification, washed with water and eluted with acetone sequentially. The eluate was concentrated to dryness under nitrogen gas flow. The dried sample was resuspended in 50% acetonitrile in water and analysed using the UPLC–MS/MS system consisting of a UPLC system (Waters) equipped with a BEH C18 column (2.1 × 100 mm, 1.7 μm, Waters) and a QTRAP 6500 mass spectrometer (AB Sciex) equipped with an electrospray (ESI) source as described previously (Xin et al. 2020) with minor modification. SLs are detected in positive multiple reaction monitoring (MRM) mode. The ESI source parameters were set as follows: ion spray voltage, 5000 V; desolvation temperature, 300°C; nebulizing gas 1, 45 psi; desolvation gas 2, 50 psi; and curtain gas, 40 psi. The selected MRM transition channels were 331.1 > 234.1 for 4DO, 334.1 > 234.1 for D_3_‐4DO.

### 4.16 Accession Numbers

Sequence data from this article can be found in the GenBank/EMBL databases under the following accession numbers: *OsWRKY53* (Os05g0343400); *D10* (Os01g0746400); *D27* (Os11g0587000); *D17* (Os04g0550600); *Os900* (Os01g0700900); *Os1400* (Os01g0701400); *Os1900* (Os02g0221900); *Os5100* (Os06g0565100); *D3* (Os06g0154200); *D53* (Os11g0104300); *OsTB1* (Os03g0706500); *OsIPA1* (Os08g0509600); *OsGT1* (Os03g0198600); *OsMOC1* (Os06g0610350); *OsMOC3* (Os04g0663600); *OsFON1* (Os06g0717200); *UBIQUITIN* (Os01g0328400); *ACTIN1* (Os03g50885); *OsSLR1* (Os03g0707600).

## Author Contributions

Q.B., X.T., Y.X., and J.T. conceived and supervised the project. J.T. and Q.B. analysed the data and wrote the article. J.T. performed most of the experiments with the help from G.Z., J.Y., Z.C., Z.H., X.J., Z.Q., Z.W., X.L., J.Y., C.L., W.L., and J.C.

## Funding

This work was supported by the National Natural Science Foundation of China, 32301913, 32441024; Natural Science Foundation of Heilongjiang Province, YQ2024C039; National Natural Science Foundation of China‐Heilongjiang Joint Fund, U23A20193; Young Scientist Group Project of Northeast Institute of Geography and Agroecology, Chinese Academy of Sciences, 2023QNXZ02.

## Ethics Statement

The authors have nothing to report.

## Consent

The authors have nothing to report.

## Conflicts of Interest

The authors declare no conflicts of interest.

## Supporting information


**Figure S1:** The *oswrky53* mutant shows increased tiller number.
**Figure S2:** Generation and identification of oswrky53‐SJ18 and oswrky53‐ZKF5 mutants.
**Figure S3:** Tillering phenotypic analysis of oswrky53 mutants in diverse backgrounds.
**Figure S4:** Expression of related tillering regulation genes in LJ11 and oswrky53.
**Figure S5:** Identification and phenotypic analysis of LJ11, oswrky53, OsTB1‐OE and oswrky53 OsTB1‐OE transgenic plants.
**Figure S6:** OsWRKY53 interacts with OsGT1 in CO‐IP assays.
**Figure S7:** OsWRKY53 interacts with OsGT1 in Pull‐down assays.
**Figure S8:** OsGT1 regulates the expression of OsTB1.
**Figure S9:** OsGT1 specifically binds to the promoter region of OsTB1.
**Figure S10:** OsGT1 enhances the DNA‐binding ability of OsWRKY53 to OsTB1.
**Figure S11:** Generation and identification of osgt1 and oswrky53 osgt1 mutants.
**Figure S12:** SL treatment leads to a significant reduction in OsWRKY53 transcript levels.
**Figure S13:** OsWRKY53 protein abundance in OsWRKY53‐FLAG ‐OE plants with or without GR24 treatment.
**Figure S14:** OsWRKY53 can not interact with D3.
**Figure S15:** OsWRKY53 interacts with D53 in Co‐IP assays.
**Figure S16:** D53 increases the protein stability of OsWRKY53 in rice protoplast.
**Figure S17:** SL promotes OsWRKY53 degradation via D53 in rice protoplast.
**Figure S18:** D53 increases the protein stability of OsWRKY53 in N. benthamiana.
**Figure S19:** D53 increases the protein stability of OsWRKY53 in cell‐free protein degradation assays.
**Figure S20:** Bioinformatic analysis of direct binding of OsWRKY53 to SL biosynthetic gene promoters.
**Figure S21:** OsWRKY53 can bind to the promoters of D10.
**Figure S22:** Agronomic trait evaluation among ZKF5 and oswrky53‐ZKF5 plants.
**Figure S23:** Functional analysis of nonsynonymous SNPs in CDS region of OsGT1.
**Figure S24:** Generation and identification of OsGT1581T(ZKF5) plant.
**Figure S25:** OsWRKY53 does not regulate D53 expression.
**Figure S26:** Agronomic trait evaluation among LJ11, oswrky53, osgt1, and oswrky53 osgt1 plants.

**Table S1:** The culm number data of 1726 rice cultivars.

**Table S2:** Primers uesd in this study.

## Data Availability

The data that support the findings of this study are available in the Supporting Information of this article.
