# Supplementary material for: OsWRKY53‐OsGT1 Module Regulates Rice Tiller Development and Is Involved in Fine‐Tuning Strigolactone Signaling
Source: Plant Biotechnol J. 2026 Feb 5;24(5):3479–93. doi: 10.1111/pbi.70578 (PMC13110158; doi:10.1111/pbi.70578)
Supplement: Supplementary file 1 — Figure S1: The oswrky53 mutant shows increased tiller number. Figure S2: Generation and identification of oswrky53‐SJ18 and oswrky53‐ZKF5 mutants. Figure S3: Tillering phenotypic analysis of oswrky53 mutants in diverse backgrounds. Figure S4: Expression of related tillering regulation genes in LJ11 and oswrky53. Figure S5: Identification and phenotypic analysis of LJ11, oswrky53, OsTB1‐OE and oswrky53 OsTB1‐OE transgenic plants. Figure S6: OsWRKY53 interacts with OsGT1 in CO‐IP assays. Figure S7: OsWRKY53 interacts with OsGT1 in Pull‐down assays. Figure S8: OsGT1 regulates the expression of OsTB1. Figure S9: OsGT1 specifically binds to the promoter region of OsTB1. Figure S10: OsGT1 enhances the DNA‐binding ability of OsWRKY53 to OsTB1. Figure S11: Generation and identification of osgt1 and oswrky53 osgt1 mutants. Figure S12: SL treatment leads to a significant reduction in OsWRKY53 transcript levels. Figure S13: OsWRKY53 protein abundance in OsWRKY53‐FLAG ‐OE plants with or without GR24 treatment. Figure S14: OsWRKY53 can not interact with D3. Figure S15: OsWRKY53 interacts with D53 in Co‐IP assays. Figure S16: D53 increases the protein stability of OsWRKY53 in rice protoplast. Figure S17: SL promotes OsWRKY53 degradation via D53 in rice protoplast. Figure S18: D53 increases the protein stability of OsWRKY53 in N. benthamiana. Figure S19: D53 increases the protein stability of OsWRKY53 in cell‐free protein degradation assays. Figure S20: Bioinformatic analysis of direct binding of OsWRKY53 to SL biosynthetic gene promoters. Figure S21: OsWRKY53 can bind to the promoters of D10. Figure S22: Agronomic trait evaluation among ZKF5 and oswrky53‐ZKF5 plants. Figure S23: Functional analysis of nonsynonymous SNPs in CDS region of OsGT1. Figure S24: Generation and identification of OsGT1581T(ZKF5) plant. Figure S25: OsWRKY53 does not regulate D53 expression. Figure S26: Agronomic trait evaluation among LJ11, oswrky53, osgt1, and oswrky53 osgt1 plants. [file PBI-24-3479-s003.pdf]

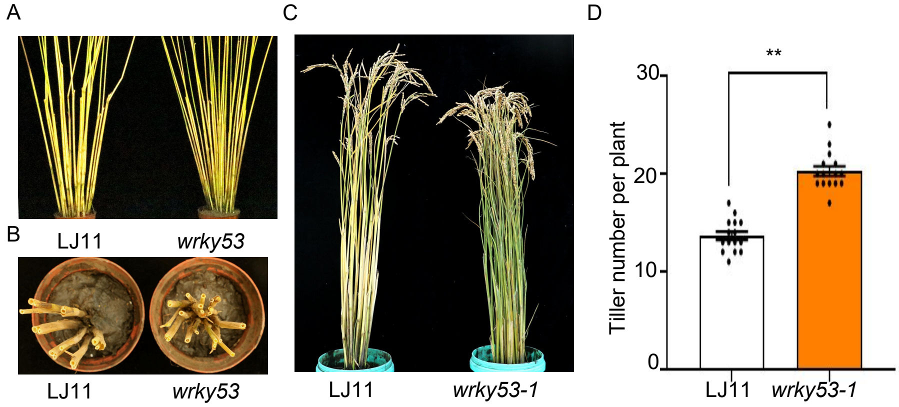
A
B
LJ11
wrky53
LJ11
wrky53
C
LJ11
wrky53-1
D
Tiller number per plant
**
30
20
10
0
LJ11
wrky53-1

SJ18 CACCGGTGCAATCCCGGCT-CAGAGGTACGACTGGAAGG

w53-SJ18 CACCGGTGCAATCCCGGCTTCAGAGGTACGACTGGAAGG

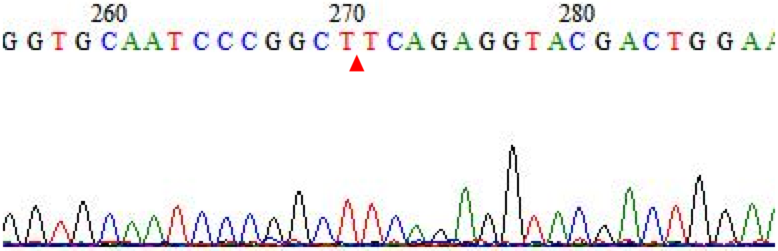

ZKF5 CACCGGTGCAATCCCGGCT-CAGAGGTACGACTGGAAGG

w53-ZKF5 CACCGGTGCAATCCCGGCTTCAGAGGTACGACTGGAAGG

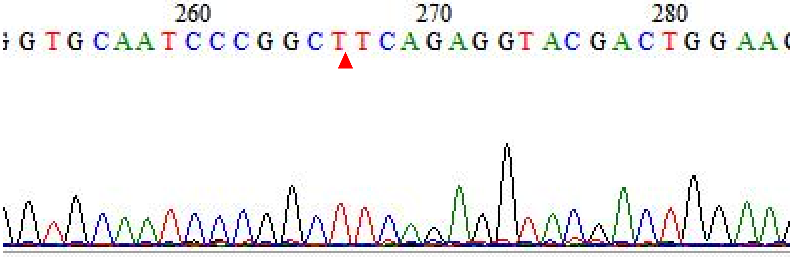

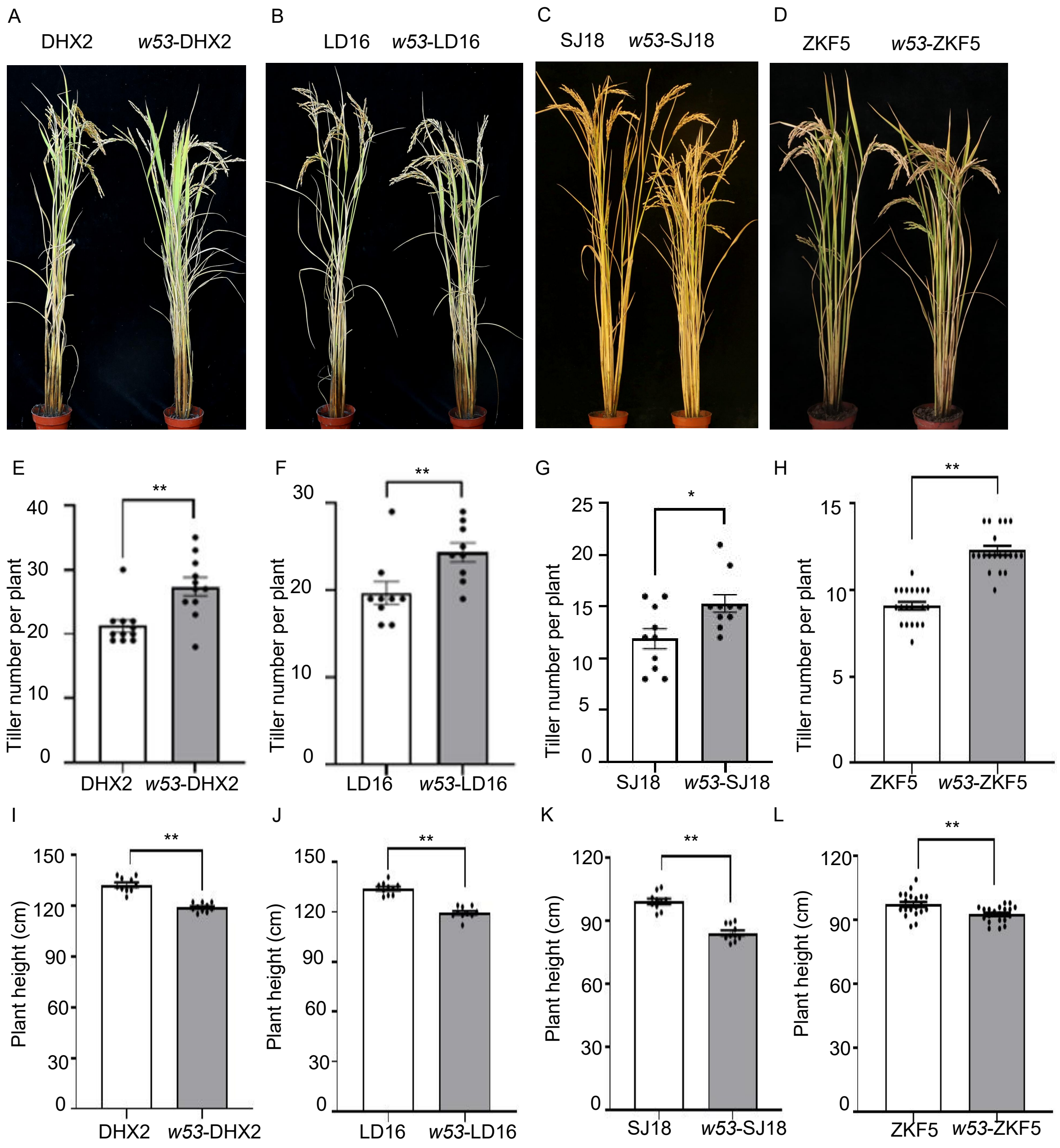
A
DHX2
w53-DHX2
B
LD16
w53-LD16
C
SJ18
w53-SJ18
D
ZKF5
w53-ZKF5
E
Tiller number per plant
DHX2
w53-DHX2
F
Tiller number per plant
LD16
w53-LD16
G
Tiller number per plant
SJ18
w53-SJ18
H
Tiller number per plant
ZKF5
w53-ZKF5
I
Plant height (cm)
DHX2
w53-DHX2
J
Plant height (cm)
LD16
w53-LD16
K
Plant height (cm)
SJ18
w53-SJ18
L
Plant height (cm)
ZKF5
w53-ZKF5

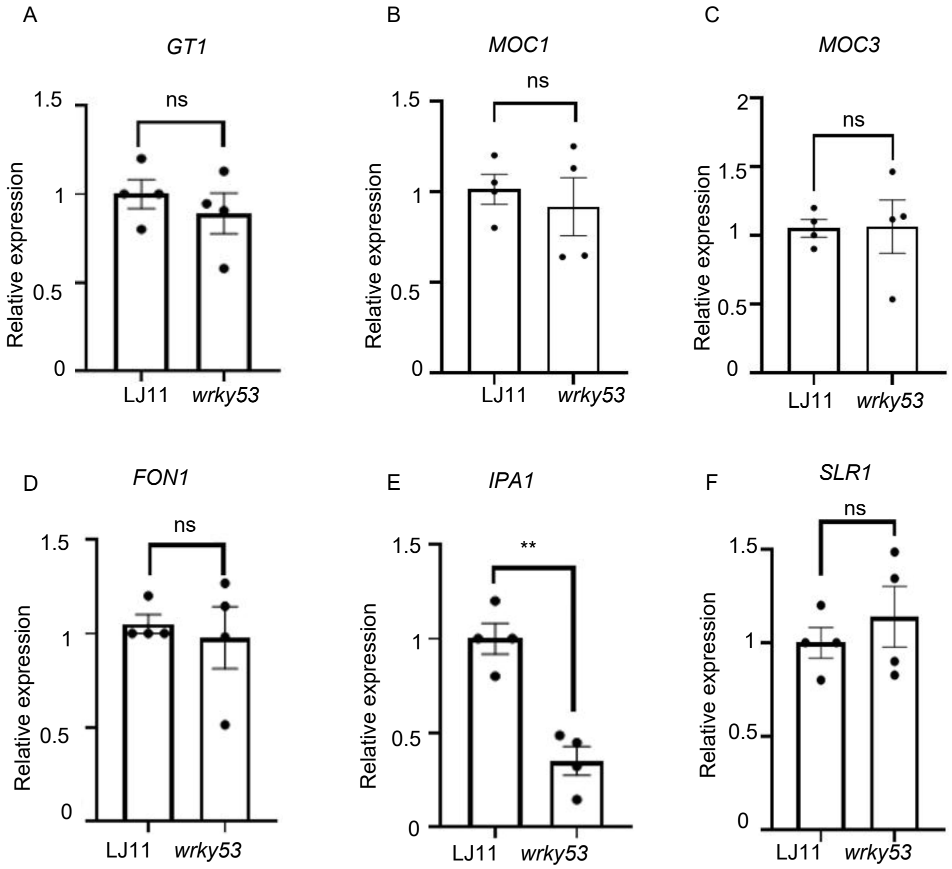
A
GT1
ns
1.5
1
0.5
0
Relative expression
LJ11
wrky53
B
MOC1
ns
1.5
1
0.5
0
Relative expression
LJ11
wrky53
C
MOC3
ns
2
1.5
1
0.5
0
Relative expression
LJ11
wrky53
D
FON1
ns
1.5
1
0.5
0
Relative expression
LJ11
wrky53
E
IPA1
**
1.5
1
0.5
0
Relative expression
LJ11
wrky53
F
SLR1
ns
1.5
1
0.5
0
Relative expression
LJ11
wrky53

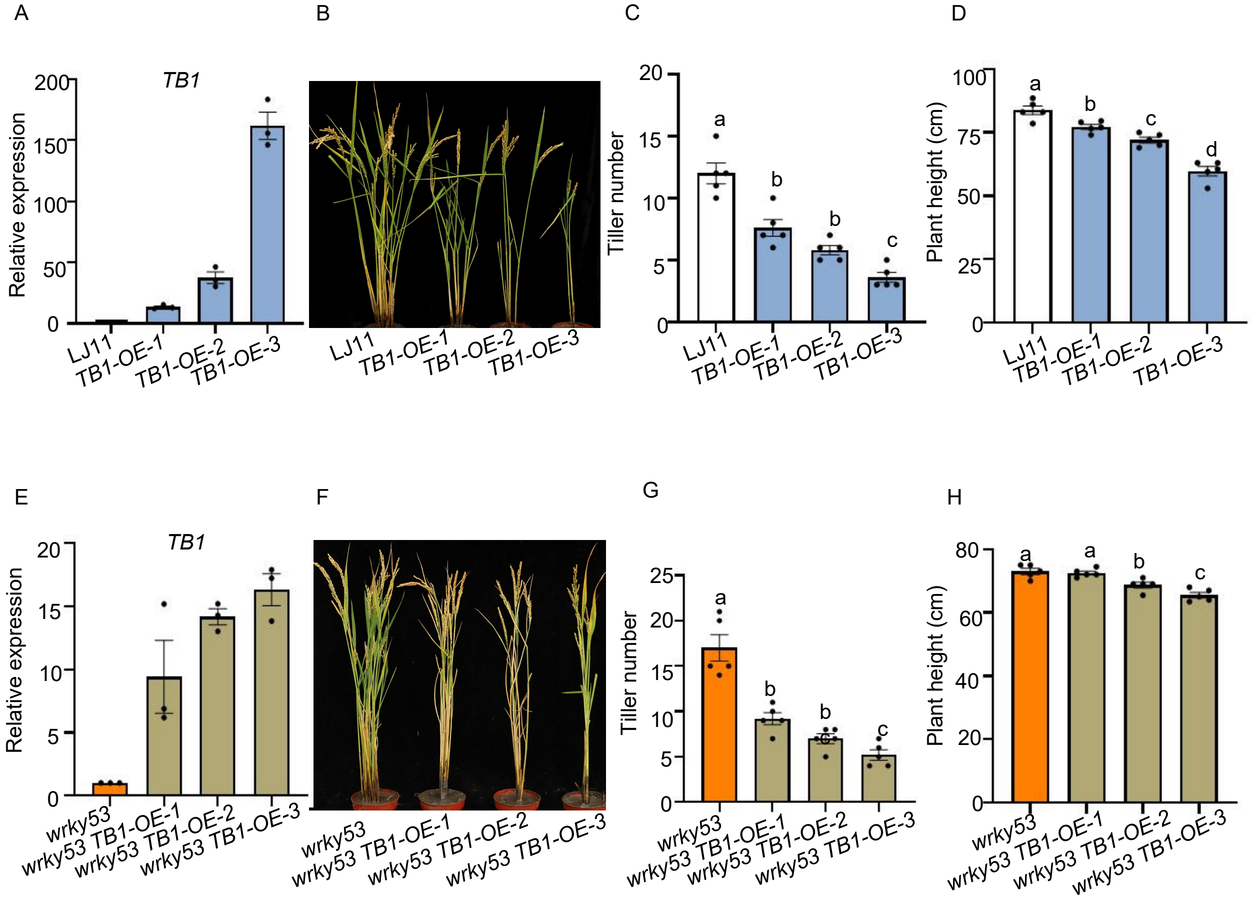

A
B
C
D
TB1
Relative expression
200
150
100
50
0
LJ11
TB1-OE-1
TB1-OE-2
TB1-OE-3
Tiller number
20
15
10
5
a
b
b
c
Plant height (cm)
100
75
25
d
E
F
G
H
wrky53
wrky53 TB1-OE-1
wrky53 TB1-OE-2
wrky53 TB1-OE-3
80
60
40

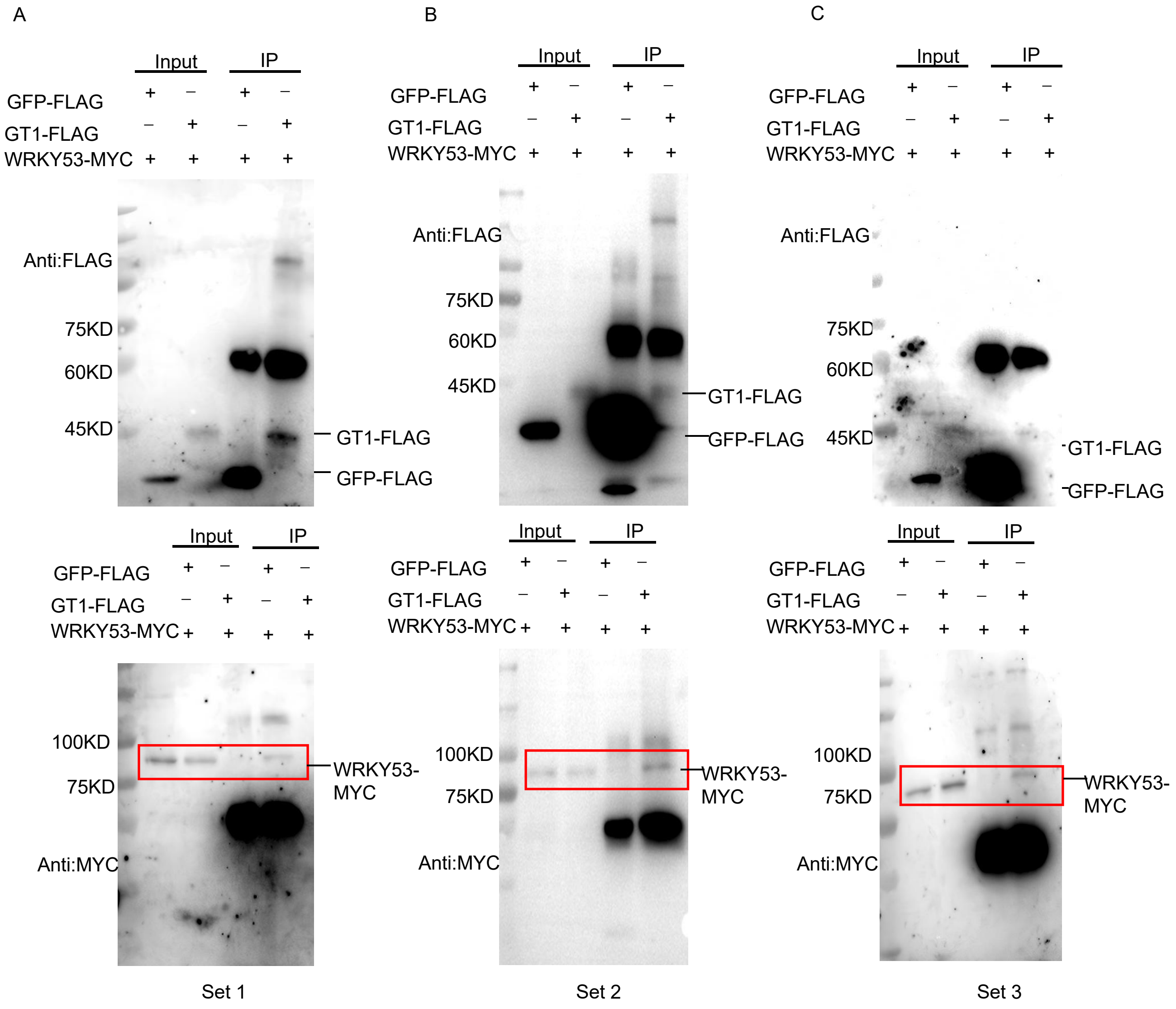
A
B
C
Input
IP
GFP-FLAG
GT1-FLAG
WRKY53-MYC
Anti:FLAG
75KD
60KD
45KD
GT1-FLAG
GFP-FLAG
100KD
WRKY53-MYC
Anti:MYC
Set 1
Set 2
Set 3

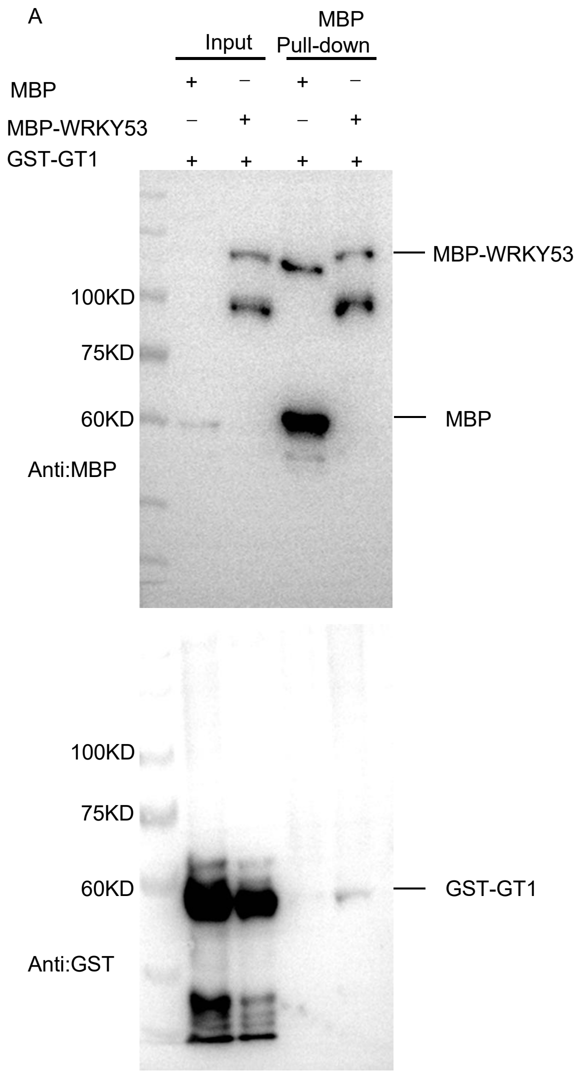
A
Input
MBP
Pull-down
MBP
MBP-WRKY53
GST-GT1
+ − + −
− + − +
+ + + +
MBP-WRKY53
100KD
75KD
60KD
MBP
Anti:MBP
100KD
75KD
60KD
GST-GT1
Anti:GST

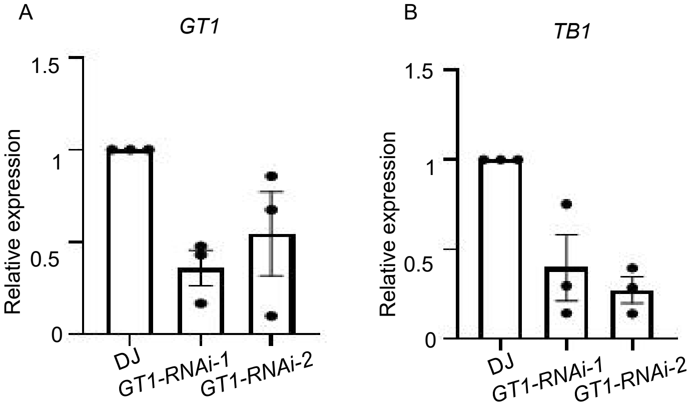
A
GT1
1.5
1
0.5
0
Relative expression
DJ
GT1-RNAi-1
GT1-RNAi-2
B
TB1
1.5
1
0.5
0
Relative expression
DJ
GT1-RNAi-1
GT1-RNAi-2

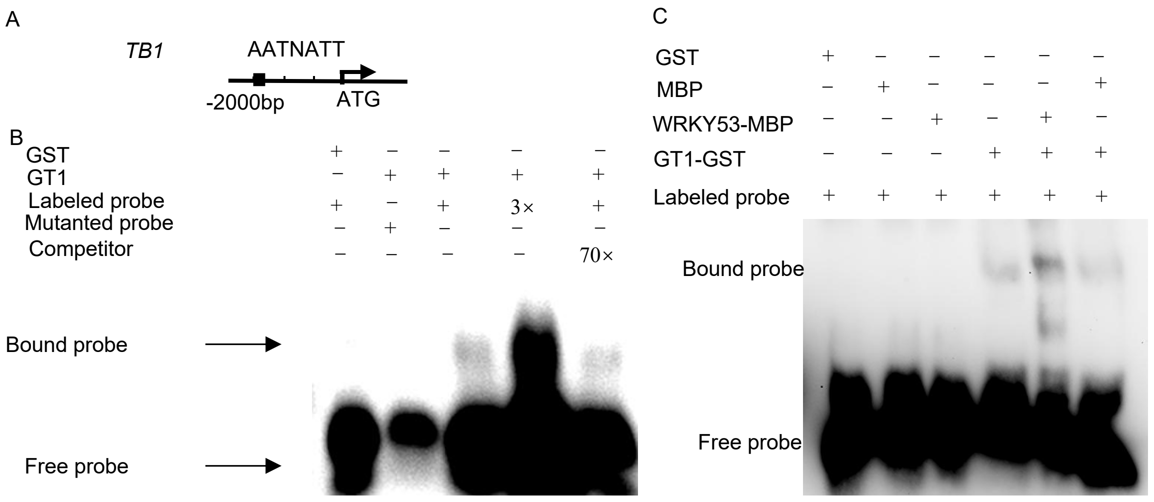
A
TB1
AATNATT
-2000bp
ATG
B
GST
GT1
Labeled probe
Mutanted probe
Competitor
3×
70×
Bound probe
Free probe
C
GST
MBP
WRKY53-MBP
GT1-GST
Labeled probe
Bound probe
Free probe

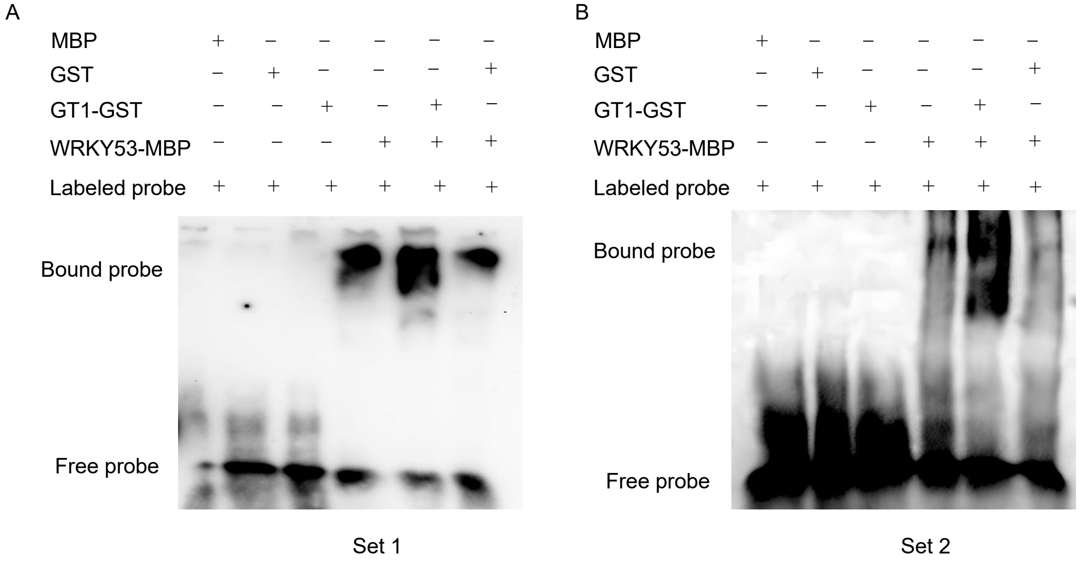
A
MBP
GST
GT1-GST
WRKY53-MBP
Labeled probe
Bound probe
Free probe
Set 1
B
MBP
GST
GT1-GST
WRKY53-MBP
Labeled probe
Bound probe
Free probe
Set 2

WT	TGAGCAGGCGAAGAAGCGGCGGCTGAGCG	CAAGCAGGTCG-CCGTCTGGTTCCAGAACCG
gt1	TGAGCAGGCGAA-AAGCGGCGGCTGAGCG	CAAGCAGGTCGCCCGTCTGGTTCCAGAACCG

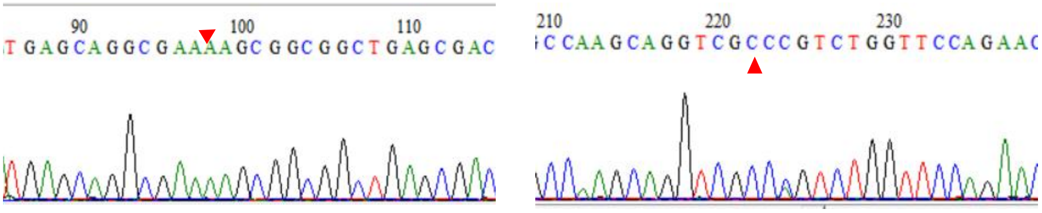

wrky53	TGAGCAGGCGAAGAAGCGGCGGCTGAGCG	CAAGCAGGTCGCCGTCTGGTTCCAGAACCG
wrky53 gt1	TGAGCAGGCGAA--AGCGGCGGCTGAGCG	CAAGCAGGTCGCCG----------AACCG

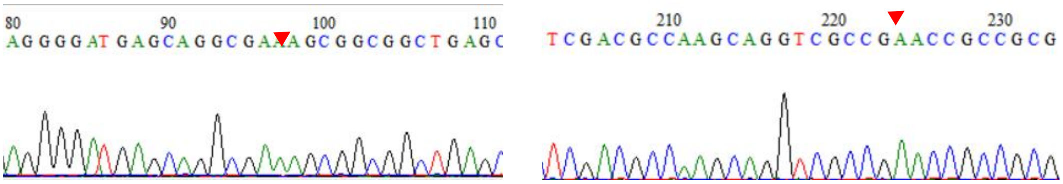

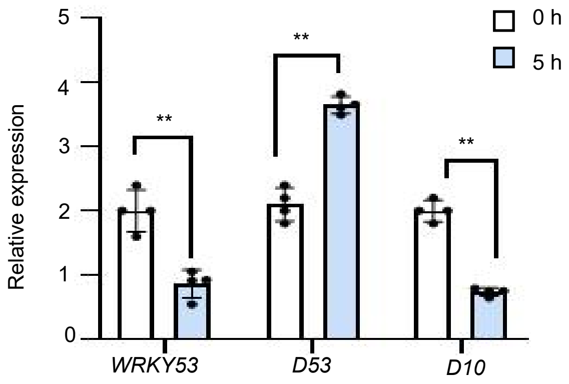

0 h
5 h
Relative expression
5
4
3
2
1
0
**
**
**
WRKY53
D53
D10

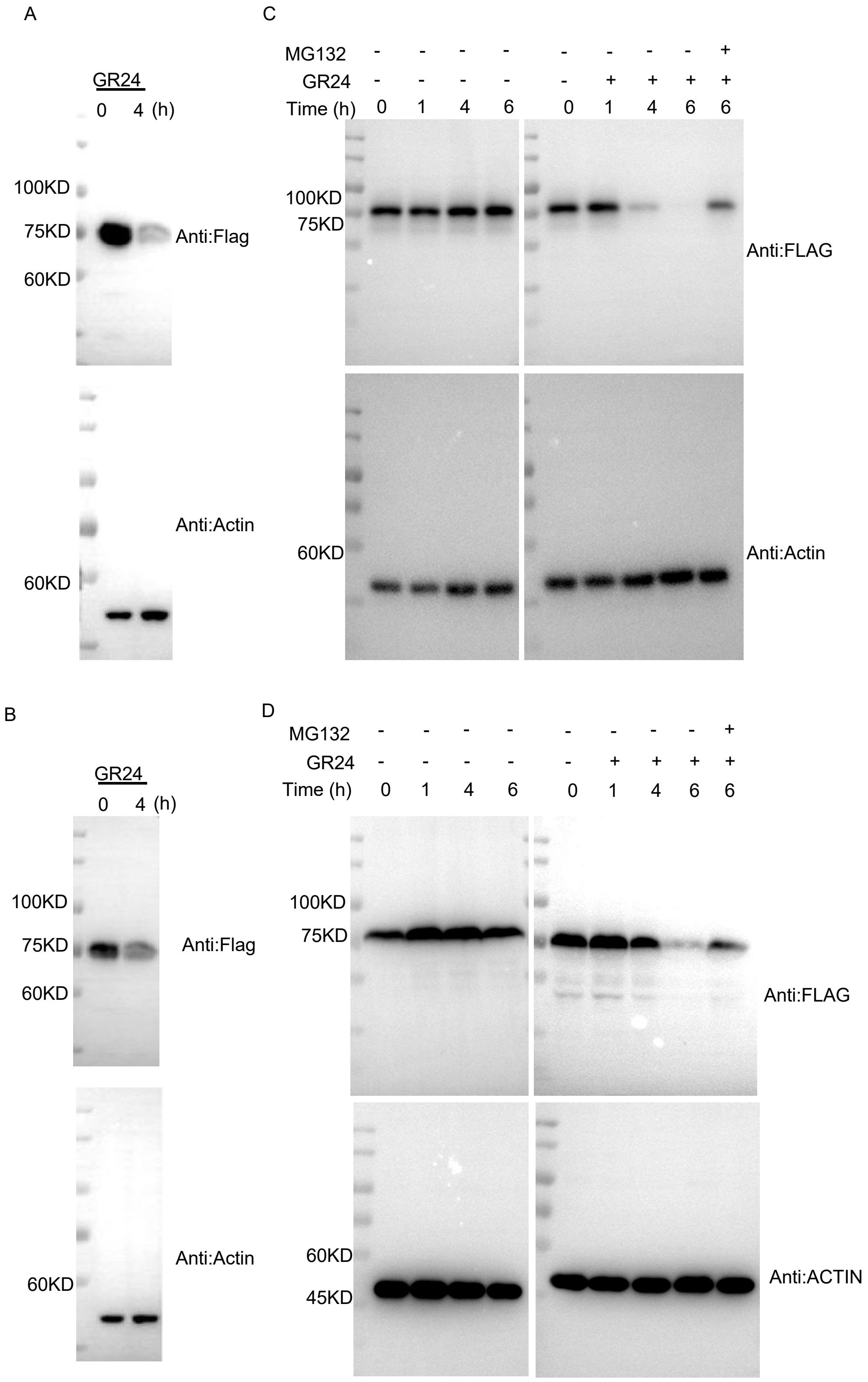
A
GR24
0
4 (h)
100KD
75KD
60KD
Anti:Flag
Anti:Actin
60KD
C
MG132
GR24
Time (h)
0 1 4 6 0 1 4 6 6
100KD
75KD
Anti:FLAG
60KD
Anti:Actin
B
GR24
0
4 (h)
100KD
75KD
60KD
Anti:Flag
Anti:Actin
60KD
D
MG132
GR24
Time (h)
0 1 4 6 0 1 4 6 6
100KD
75KD
Anti:FLAG
60KD
45KD
Anti:ACTIN

A

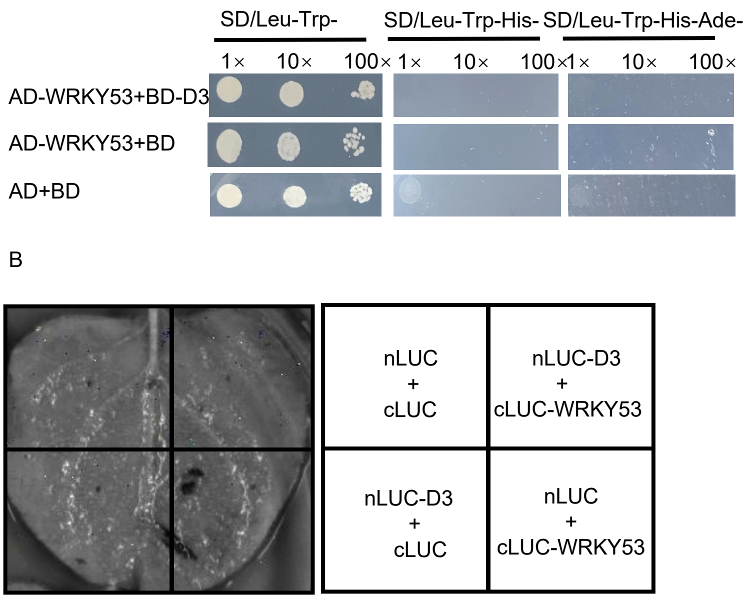
SD/Leu-Trp-
SD/Leu-Trp-His-
SD/Leu-Trp-His-Ade-
1× 10× 100× 1× 10× 100× 1× 10× 100×
AD-WRKY53+BD-D3
AD-WRKY53+BD
AD+BD
B
nLUC + cLUC
nLUC-D3 + cLUC-WRKY53
nLUC-D3 + cLUC
nLUC + cLUC-WRKY53

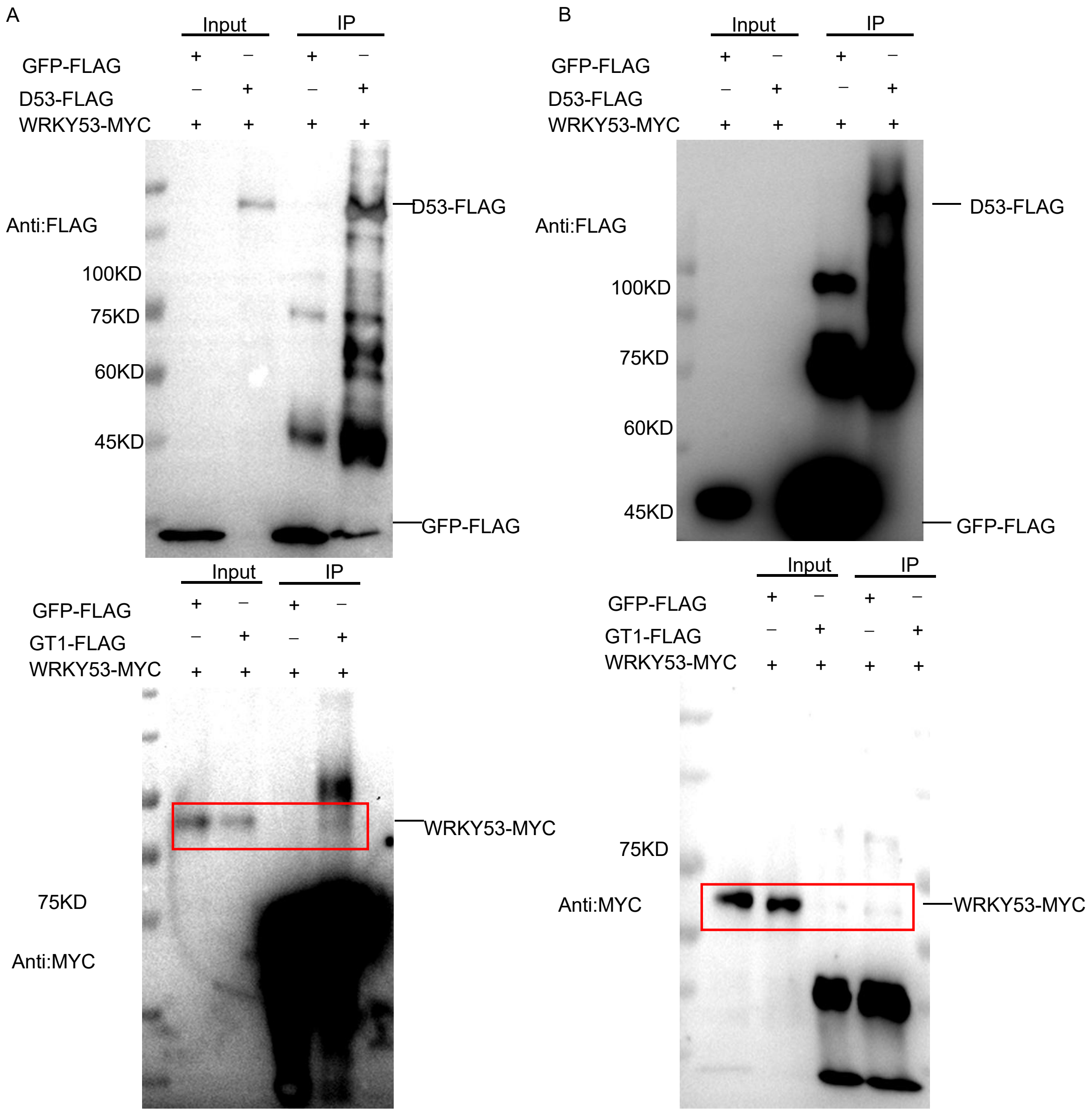
A
Input
IP
GFP-FLAG + − + −
D53-FLAG − + − +
WRKY53-MYC + + + +
Anti:FLAG
D53-FLAG
100KD
75KD
60KD
45KD
GFP-FLAG
Input
IP
GFP-FLAG + − + −
GT1-FLAG − + − +
WRKY53-MYC + + + +
WRKY53-MYC
75KD
Anti:MYC
B
Input
IP
GFP-FLAG + − + −
D53-FLAG − + − +
WRKY53-MYC + + + +
Anti:FLAG
D53-FLAG
100KD
75KD
60KD
45KD
GFP-FLAG
Input
IP
GFP-FLAG + − + −
GT1-FLAG − + − +
WRKY53-MYC + + + +
75KD
Anti:MYC
WRKY53-MYC

Set 1

Set 2

A

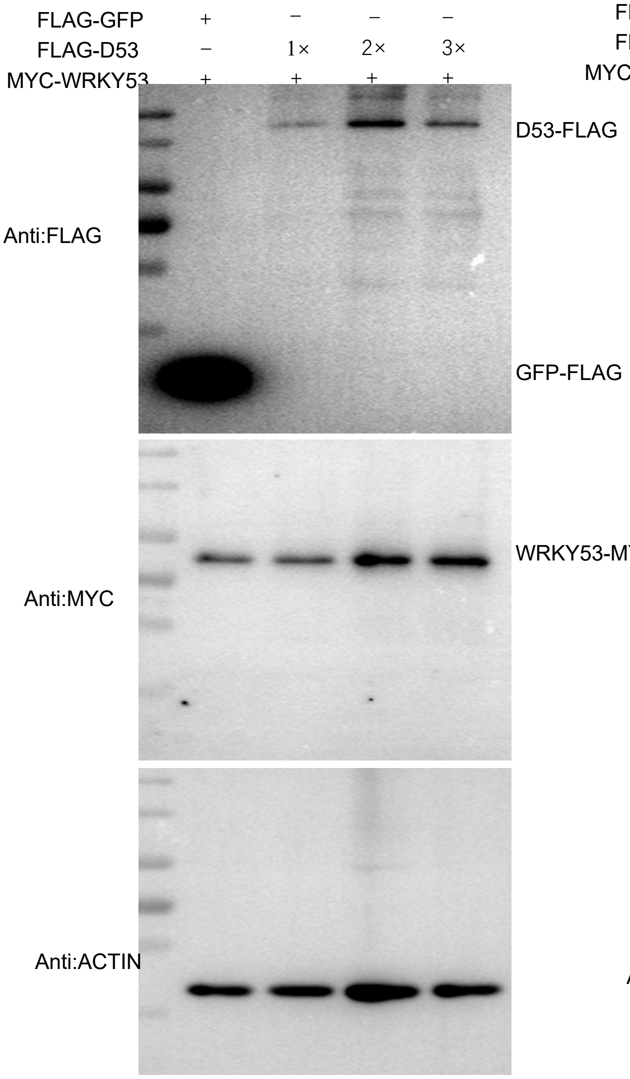
FLAG-GFP + − − −
FLAG-D53 − 1× 2× 3×
MYC-WRKY53 + + + +
D53-FLAG
Anti:FLAG
GFP-FLAG
WRKY53-MYC
Anti:MYC
Anti:ACTIN

Set 1

B

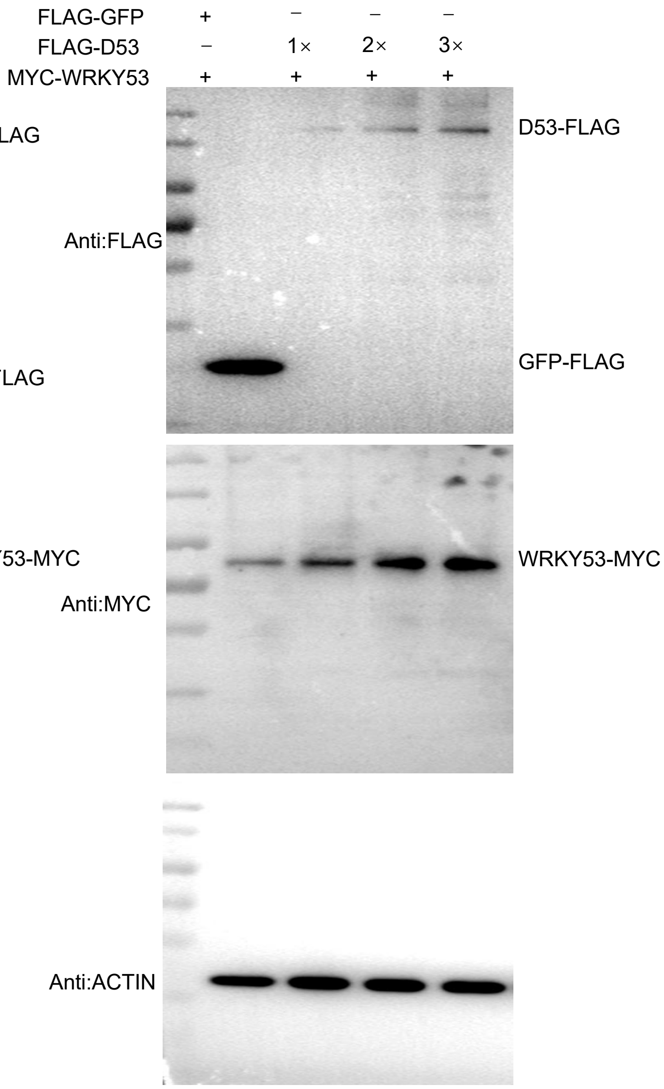
FLAG-GFP + − − −
FLAG-D53 − 1× 2× 3×
MYC-WRKY53 + + + +
D53-FLAG
Anti:FLAG
GFP-FLAG
WRKY53-MYC
Anti:MYC
Anti:ACTIN

Set 2

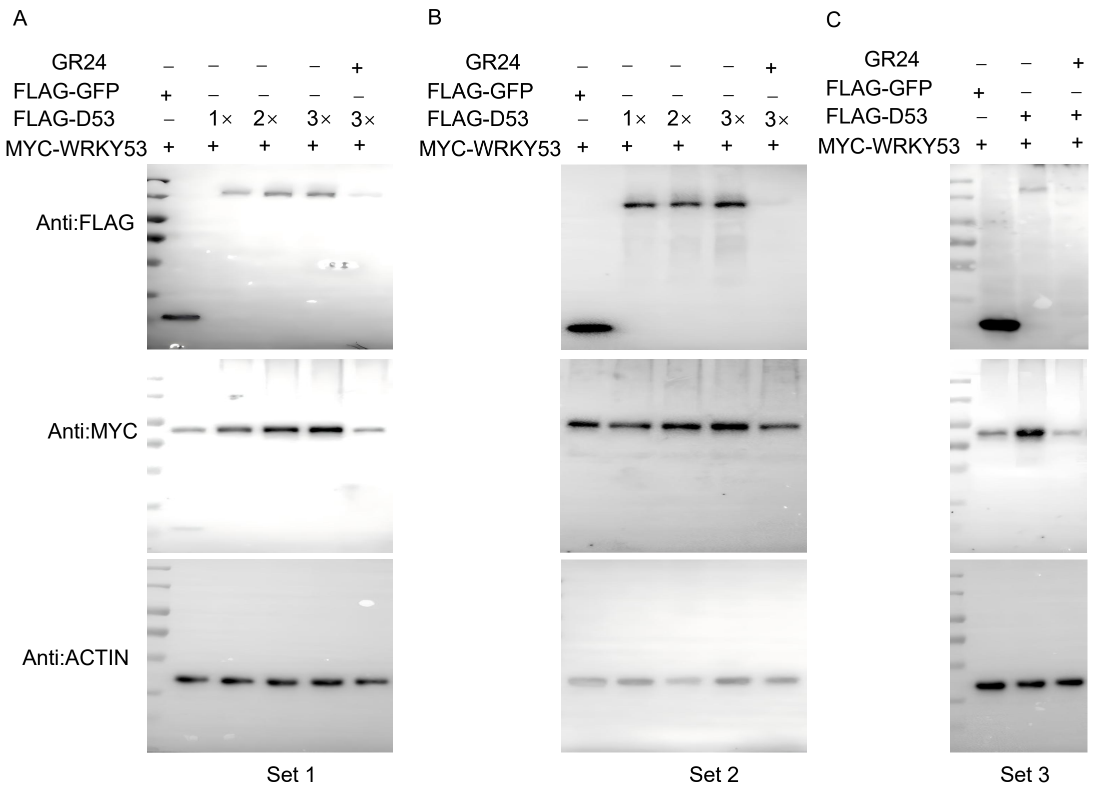
A
B
C
GR24
FLAG-GFP
FLAG-D53
MYC-WRKY53
1×
2×
3×
Anti:FLAG
Anti:MYC
Anti:ACTIN
Set 1
Set 2
Set 3

A

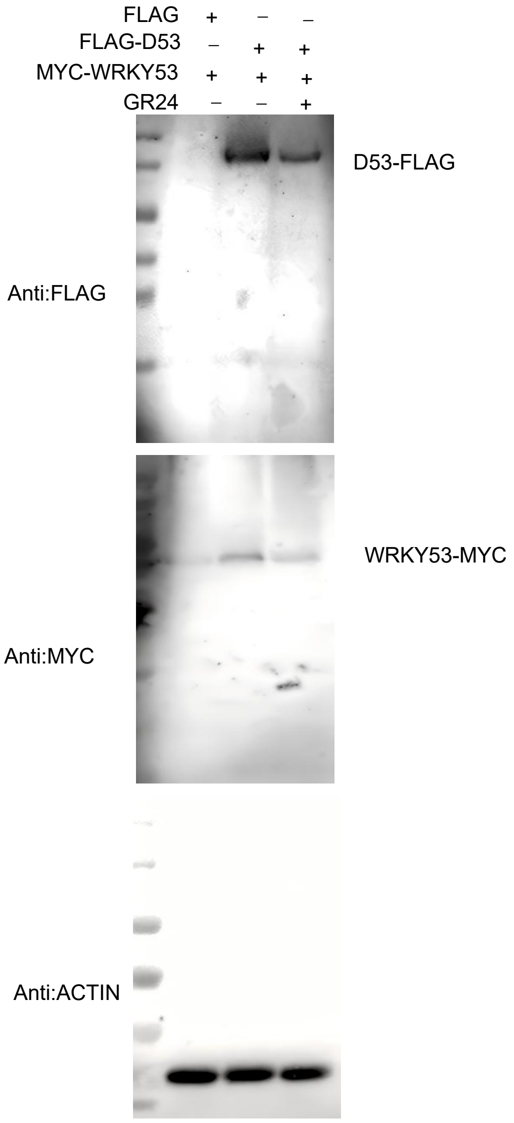
FLAG + – –
FLAG-D53 – + +
MYC-WRKY53 + + +
GR24 – – +
D53-FLAG
Anti:FLAG
WRKY53-MYC
Anti:MYC
Anti:ACTIN

Set 1

B

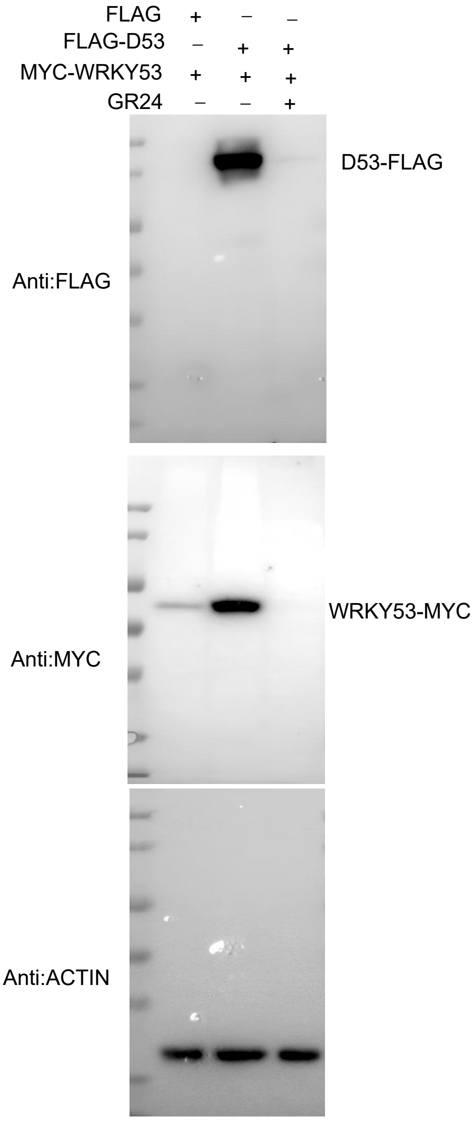
FLAG + – –
FLAG-D53 – + +
MYC-WRKY53 + + +
GR24 – – +
D53-FLAG
Anti:FLAG
WRKY53-MYC
Anti:MYC
Anti:ACTIN

Set 2

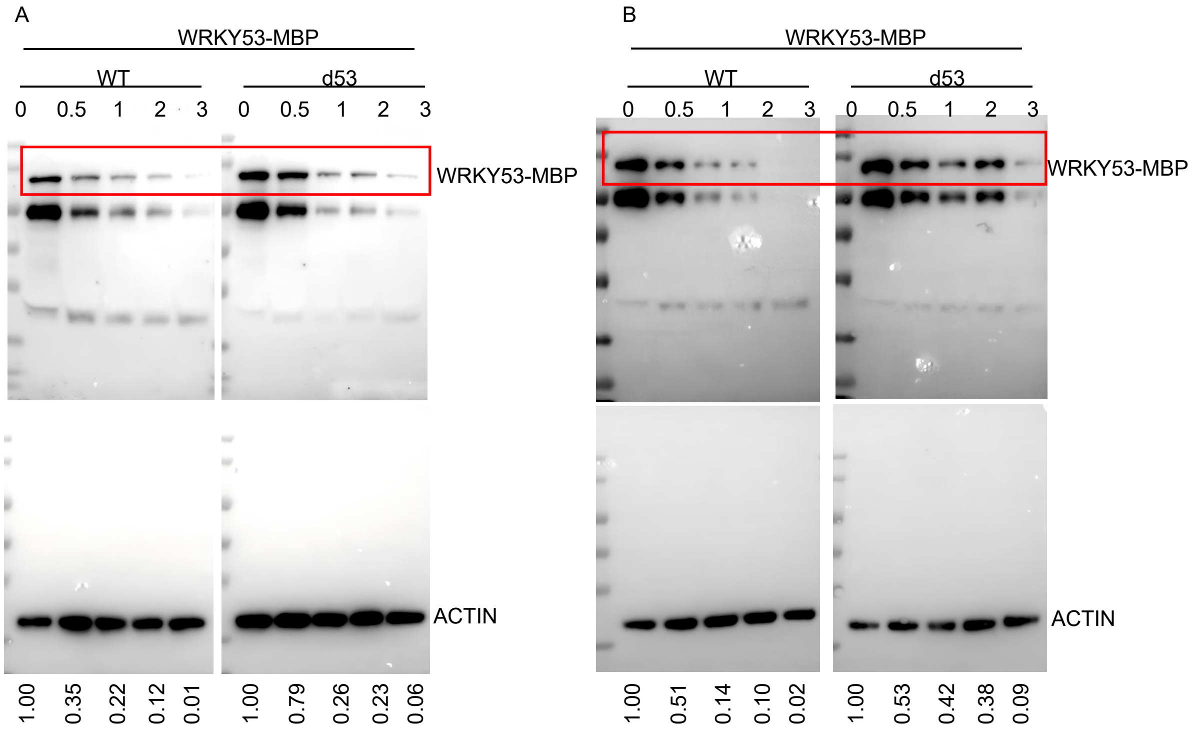
A
WRKY53-MBP
WT
d53
0 0.5 1 2 3 0 0.5 1 2 3
WRKY53-MBP
ACTIN
1.00 0.35 0.22 0.12 0.01 1.00 0.79 0.26 0.23 0.06
B
WRKY53-MBP
WT
d53
0 0.5 1 2 3 0 0.5 1 2 3
WRKY53-MBP
ACTIN
1.00 0.51 0.14 0.10 0.02 1.00 0.53 0.42 0.38 0.09

Set 1

Set 2

D10

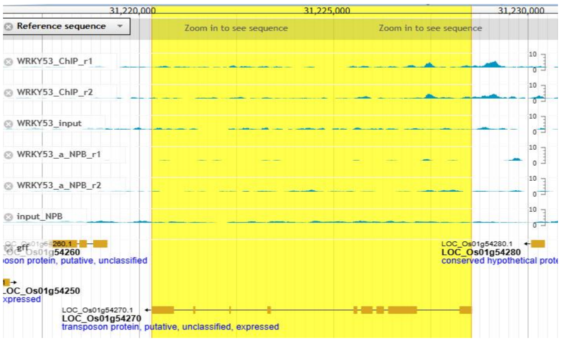

D17

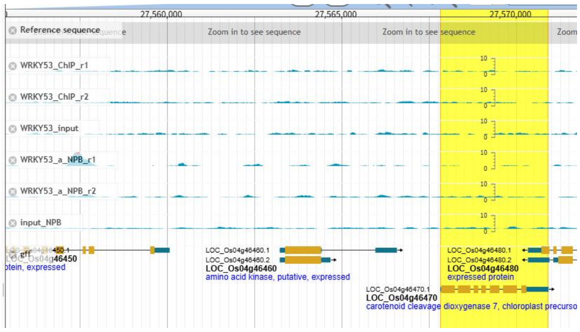

MAX1-900

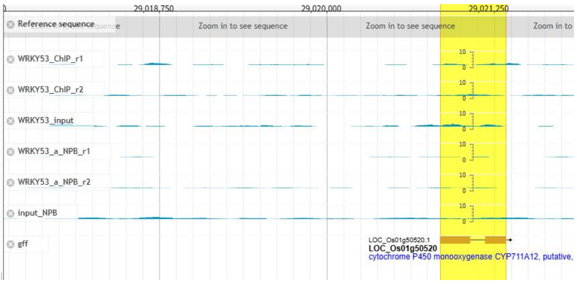

MAX1-1400

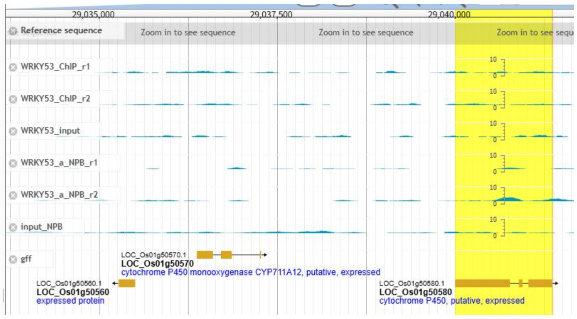

MAX1-5100

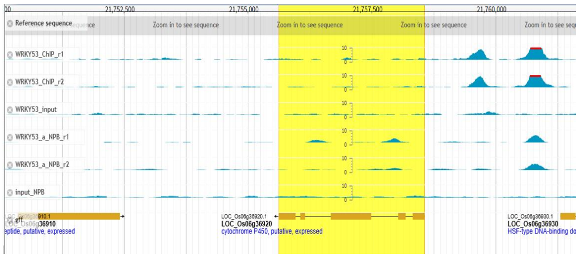

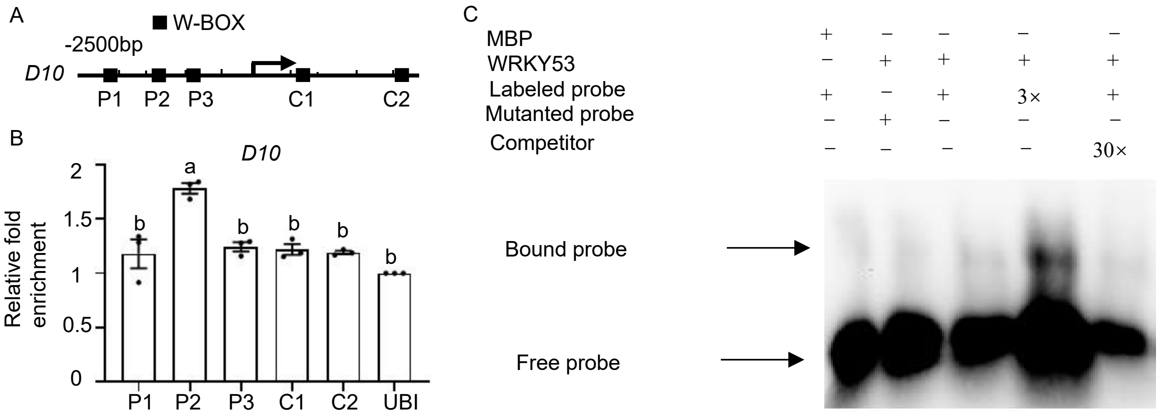
A
W-BOX
-2500bp
D10
P1
P2
P3
C1
C2
B
D10
Relative fold enrichment
2
1.5
1
0.5
0
a
b
P1
P2
P3
C1
C2
UBI
C
MBP
WRKY53
Labeled probe
Mutanted probe
Competitor
3×
30×
Bound probe
Free probe

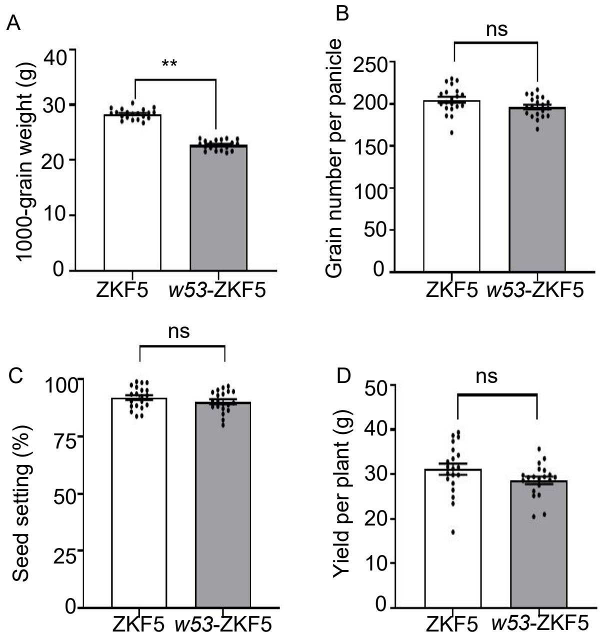

A
1000-grain weight (g)
**
0
10
20
30
40
ZKF5
w53-ZKF5
B
ns
Grain number per panicle
0
50
100
150
200
250
ZKF5
w53-ZKF5
C
ns
Seed setting (%)
0
25
50
75
100
ZKF5
w53-ZKF5
D
ns
Yield per plant (g)
0
10
20
30
40
50
ZKF5
w53-ZKF5

Var ID	Chrom	Position	Primary Allele	Secondary Allele	Effect snpEff and CooVar
vg0305181154 (J)	chr03	5181154	A	G	frameshift_variant/ nonsynonymous_codon/ missense_variant LOC_Os03g10210.1 Alt: A| missense_variant MODERATE(snpEff)/nonsynonymous_codon(CooVar)LOC_Os03g10210.1 Alt: DEL/frameshift_variant(CooVar)The average chromatin accessibility score: 85.463; most accessible tissue: Minghui63 panicle, score: 96.467
vg0305181957 (J)	chr03	5181957	T	A	frameshift_variant/ nonsynonymous_codon/ missense_variant LOC_Os03g10210.1 Alt: T| missense_variant MODERATE(snpEff)/nonsynonymous_codon(CooVar)LOC_Os03g10210.1 Alt: DEL/frameshift_variant(CooVar)The average chromatin accessibility score: 84.207; most accessible tissue: Minghui63 panicle, score: 97.431

ZKF5 TGACGTACCACCCGGCGCTGGCGGGGC

GT1^{581T} TGACGTACCACCCGGTGCTGGCGGGGC

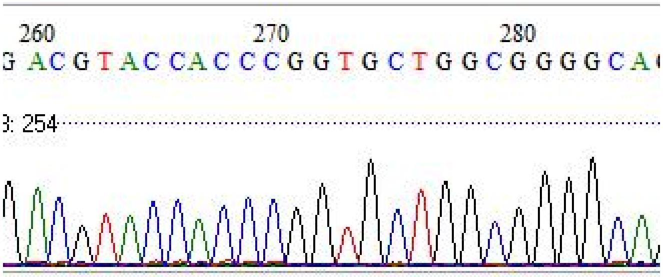

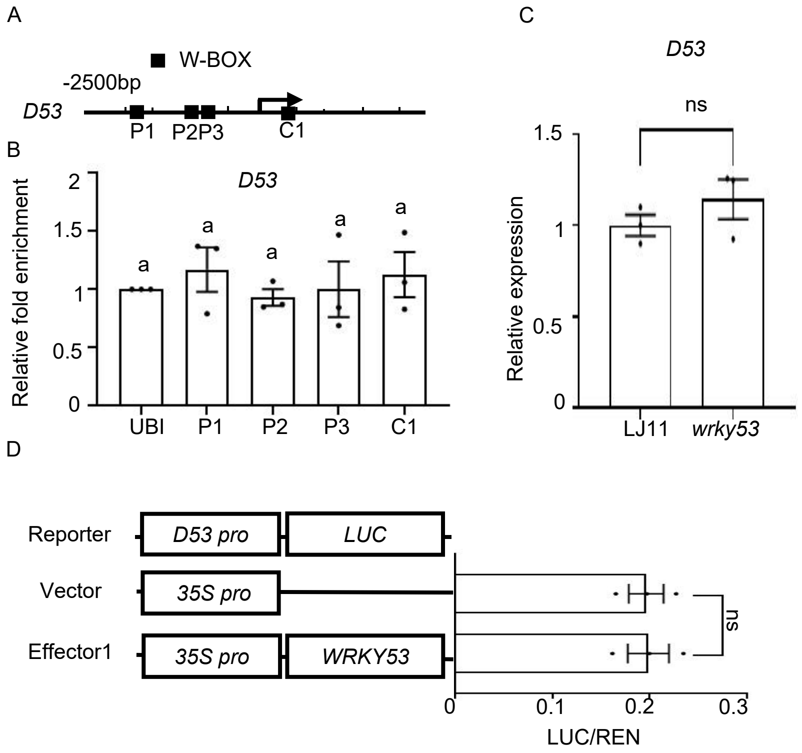
A
W-BOX
-2500bp
D53
P1
P2P3
C1
B
D53
Relative fold enrichment
2
1.5
1
0.5
0
a
a
a
a
a
UBI
P1
P2
P3
C1
C
D53
ns
Relative expression
1.5
1
0.5
0
LJ11
wrky53
D
Reporter
D53 pro
LUC
Vector
35S pro
Effector1
35S pro
WRKY53
ns
0
0.1
0.2
0.3
LUC/REN

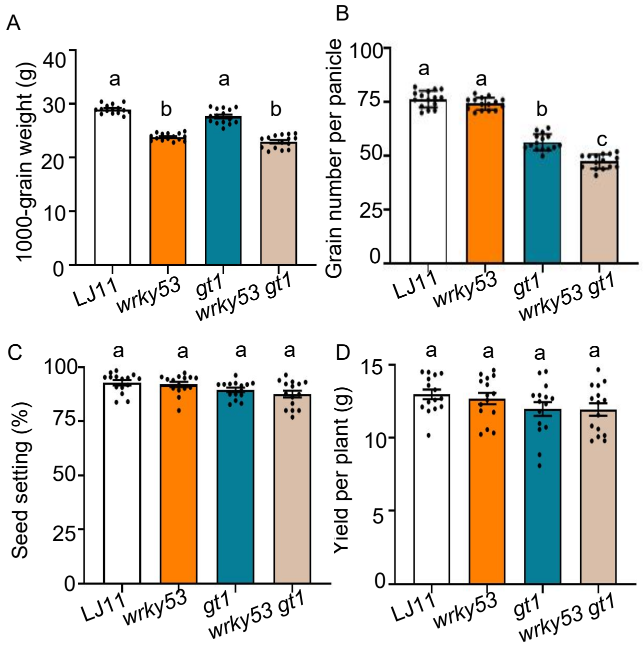
A
1000-grain weight (g)
40
30
20
10
0
a
b
a
b
LJ11
wrky53
gt1
wrky53 gt1
B
Grain number per panicle
100
75
50
25
0
a
a
b
c
LJ11
wrky53
gt1
wrky53 gt1
C
Seed setting (%)
100
75
50
25
0
a
a
a
a
LJ11
wrky53
gt1
wrky53 gt1
D
Yield per plant (g)
15
10
5
0
a
a
a
a
LJ11
wrky53
gt1
wrky53 gt1
